# DMAP-stabilized bis(silyl)silylenes as versatile synthons for organosilicon compounds†

**DOI:** 10.1039/c9ra10628f

**Published:** 2020-01-21

**Authors:** Richard Holzner, Dominik Reiter, Philipp Frisch, Shigeyoshi Inoue

**Affiliations:** Department of Chemistry, WACKER-Institute of Silicon Chemistry and Catalysis Research Center Lichtenbergstraße 4 85748 Garching bei München Germany s.inoue@tum.de

## Abstract

DMAP-stabilized silylenes 1a–c are obtained from the reductive debromination of the corresponding dibromosilanes in the presence of DMAP. Their distinctly different thermal isomerization reactions *via* C–H bond activation, dearomative ring expansion and silyl migration are discussed. Furthermore, complexes 1 dissociate at elevated temperatures, providing the corresponding free silylenes *in situ*, which are even capable of single-site activation of H_2_. Additionally, a potassium-substituted silicon-centered radical 2 is isolated from overreduction of (^*t*^Bu_3_Si)_2_SiBr_2_.

## Abstract 2

DMAP-stabilized silylenes 1a–c, which are convenient, room temperature stable synthetic equivalents for the corresponding highly reactive free bis(silyl)silylenes are reported.

## Graphical abstract

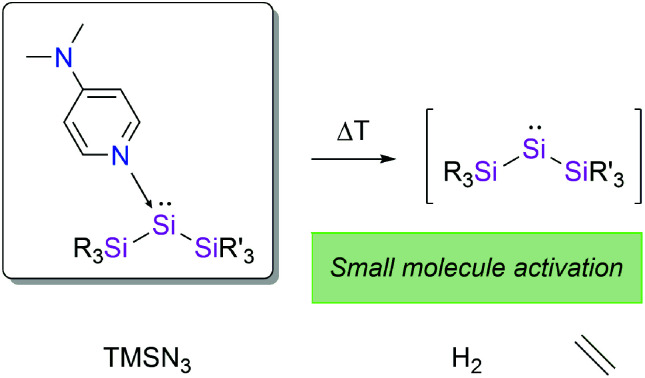

## Introduction

**Fig. 1:**
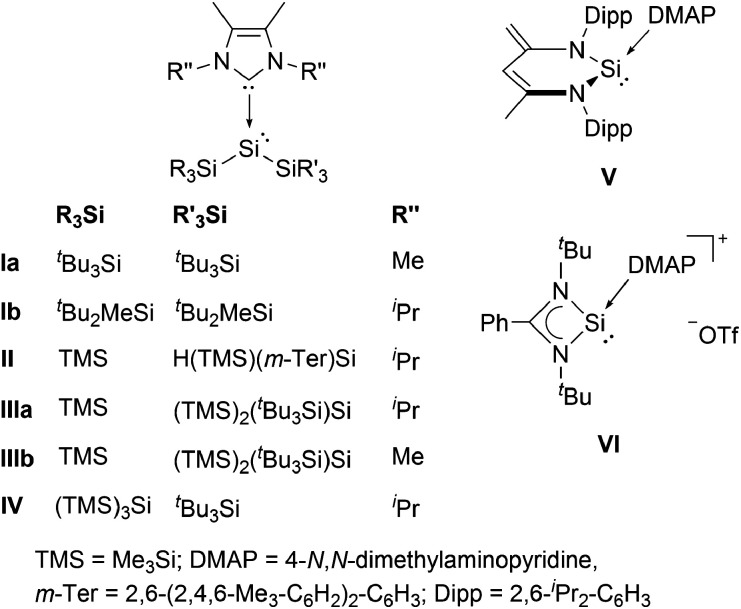
Acyclic NHC-stabilized bis(silyl)silylenes I–IV and low-coordinate silicon DMAP complexes V and VI.

**Scheme 1:**
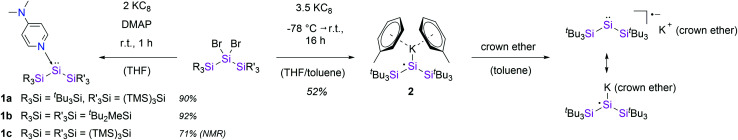
Synthesis of DMAP-stabilized silylenes 1a–c and silyl radical 2.

Silylenes (R_2_Si:), the heavier congeners of carbenes (R_2_C:) have attracted much attention in modern main group chemistry in recent years.^1^ The substituents R can either be monodentate, or cyclic, bidentate ligands, as in the case of the extensively studied class of *N*-heterocyclic silylenes (NHSis). In general, silylenes possess a lone pair of electrons and an empty 3p_*z*_ orbital and can therefore display ambiphilic reaction behaviour both as Lewis bases and Lewis acids. This particular reactivity profile even enables the facile activation of small molecules.^2^ Thus, silylenes are considered to be promising candidates for metal-free catalysis.^2^ In contrast to carbenes, however, the singlet ground state is energetically favoured for almost all reported silylenes. The two sole exceptions are transient silylenes bearing two bulky and strongly electropositive supersilyl (^*t*^Bu_3_Si) substituents, or both supersilyl groups and alkali metal substituents. However, these species were only generated and analyzed *in situ* at temperatures below 15 K.^3^ These reports already underline the peculiarity of bis(silyl)silylenes. In fact, no room temperature stable, two-coordinate derivative has been isolated to date. In all synthetic attempts the extremely reactive bis(silyl)silylene was not stable and either silyl migration^4^ or C–H bond activation occurred, even at low temperatures.^3*a*^ Very recently, we presented a bis(silyl)silylene that undergoes reversible isomerization to the corresponding tetra(silyl)disilene.^5^ Although this compound is relatively stable, it eventually decomposes *via* insertion of the silylene moiety into a C–H bond of a substituent. A convenient method to stabilize silylenes is to control their excessive electrophilicity by coordination of a Lewis base, as already recognized by Tokitoh and co-workers in 1997.^6^ In fact, electron donation from *N*-heterocyclic carbenes (NHCs) was the only way so far to isolate bis(silyl)silylenes.^7^ Sekiguchi *et al.* successfully employed this approach and obtained the NHC-stabilized silylenes I (Fig. 1).^8^ Lately, several additional examples of acyclic bis(silyl)silylene NHC complexes were reported by Cowley (II)^9^ and by our group (III and IV).^5^ Besides those acyclic representatives, the groups of Scheschkewitz^10^ and Lips^11^ synthesized NHC-stabilized silylenes with the low-coordinate silicon center being embedded in a three-membered silicon cycle. Although electron-donation of NHCs to the vacant p-orbital of silylenes is an effective method to allow isolation of these compounds, it brings the downside of a significantly reduced reactivity. Accordingly, none of the examples listed is capable of activating small molecules such as dihydrogen. Therefore, a weaker donor–acceptor interaction is necessary to achieve a balance between reactivity and stability of the respective bis(silyl)silylene compounds. 4-*N*,*N*-Dimethylaminopyridine (DMAP) is a much weaker Lewis base, compared to NHCs and was already applied by the groups of Driess^12^ and So^13^ to isolate the low-coordinate silicon donor–acceptor complexes V and VI. Thus, we envisioned DMAP to be a suitable Lewis base, strong enough to stabilize elusive bis(silyl)silylenes, yet weak enough to partially maintain their reactivity. Very recently, we reported the first acyclic bis(silyl)silylene–DMAP adduct 1a (*cf.*Scheme 1).^5^

Herein, we extend this class of donor-stabilized, highly reactive bis(silyl)silylenes. Decomposition pathways and reactivity of these novel silylenes are presented and discussed in detail. Additionally, we report the synthesis and characterization of the potassium-substituted silyl radical 2.

## Results and discussion

### Synthesis of novel DMAP–silylene complexes 1 and radical 2

**Fig. 2:**
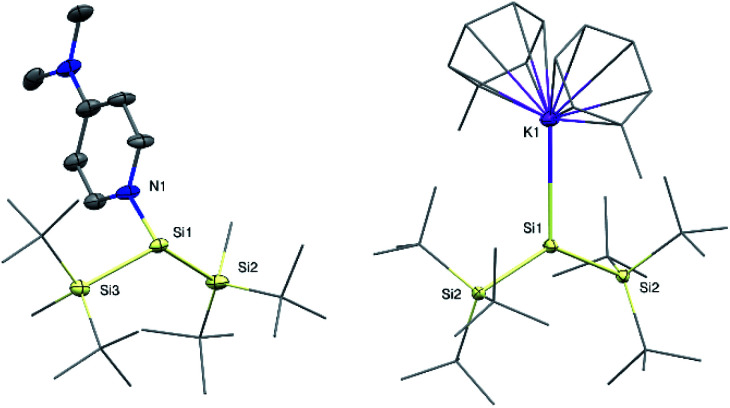
Molecular structures of silylene 1b (left) and silyl radical 2 (right) with thermal ellipsoids drawn at the 30% probability level. Hydrogen atoms are omitted for clarity. Selected bond lengths [Å] and angles [°]: 1b: Si1–N1 1.937(5), Si1–Si2 2.390(3), Si1–Si3 2.378(3), Si2–Si1–Si3 123.1(1), Si2–Si1–N1 96.2(2), Si3–Si1–N1 98.8(2); 2: Si1–Si2 2.3936(14), Si1–K1 3.315(2), K1–Si1–Si2 114.91(2), Si2–Si1–Si2* 130.19(3).

In an approach analogue to the synthesis of 1a, we obtained the donor-stabilized bis(silyl)silylene 1b from the reductive debromination of the corresponding dibromosilane with KC_8_ in presence of DMAP (Scheme 1). Silylene 1b was obtained as red-brown crystals in excellent yield (92%) and fully characterized. Neither the formation of any decomposition products, nor of the disilene (^*t*^Bu_2_MeSi)_2_Si=Si(Si^*t*^Bu_2_Me)_2_ ^14^ was observed during the synthesis. Compared to compound 1a, the ^29^Si NMR signal of the silylene Si atom in 1b is slightly upfield-shifted to 61.5 ppm (68.8 ppm in 1a). Single crystal X-ray diffraction (SC-XRD) analysis revealed a Si:–N^DMAP^ bond length in compound 1b of 1.937(5) Å (Fig. 2). This value is essentially identical to that in 1a (1.942(2) Å)^5^ and clearly within the range of previously reported low-coordinate silicon–DMAP donor–acceptor complexes (1.84–2.01 Å).^9,12,13,15^ The high degree of pyramidalization around the silylene center in 1b (sum of bond angles Σ*θ* = 318.1°) results from the stereo-chemically active electron lone pair and also compares very well to 1a (Σ*θ* = 318.7°).^5^

Additionally, the steric hindrance of the silylene center was increased by introducing bulky hypersilyl groups ((TMS)_3_Si), resulting in complex 1c. Compound 1c, which is the first stable bis(hypersilyl)silylene species, was identified by the characteristic ^29^Si NMR signal of the low-coordinate silicon nucleus (72.5 ppm), similar to the resonances of 1a and 1b.^5^ Remarkably, in this case, the facile TMS-migrations were prevented by the coordination of DMAP and the silylene could be stabilized successfully. In sharp contrast, we were not able to isolate the bis(hypersilyl)silylene moiety with NHCs. This result underlines the difference in reactivity between NHCs and the weaker Lewis base DMAP. Unfortunately, the reaction was accompanied by the by-product formation of hexakis(trimethylsilyl)trisilirane (4) and Si(TMS)_4_, reflecting the high propensity of hypersilyl groups towards TMS-group migrations.

Despite several attempts, we were not able to isolate the DMAP-stabilized bis(supersilyl) silylene (^*t*^Bu_3_Si)_2_Si: ← DMAP with the same approach used for the syntheses of 1. Even at low temperatures, the reduction of the corresponding dibromosilane only afforded the decomposition product of the free silylene (disiletane from C–H bond activation).^3*a*^ With an excess of 3.5 equivalents of KC_8_, however the potassium-substituted silyl radical 2 was generated, even in the presence of DMAP. The solid state structure of 2 was unambiguously determined by SC-XRD analysis (Fig. 2). Silyl radical 2 exhibits a completely planar geometry (sum of bond angles Σ*θ* = 360.0°) which is typical for alkali metal-substituted silyl radicals.^16^ The Si–K bond distance (3.315(2) Å) is in the same range as observed in four-coordinate potassium silanides, such as hypersilyl potassium (3.352(4) Å).^4*a*^ Thus compound 2 is clearly a contact ion pair in the solid state. Unfortunately, 2 is extremely sensitive and decomposes in toluene solution. Therefore, no satisfactory spectroscopic data was obtained. After synthesis in absence of DMAP and stabilization by crown ether (18-C-6) however, we were able to obtain an EPR spectrum which contains a signal with a *g* value of 2.0056 and a hyperfine coupling *a*(α-^29^Si) = 2.92 mT (see ESI, Fig. S7†). Coupling with the β-^29^Si nuclei was not observable. This *g* value is in the same range, as it was reported for other alkali metal-substituted silyl radicals.^16,17^ Furthermore, no signal splitting from coupling of the unpaired electron with the K nucleus was observed. Presumably, in solution, compound 2 in presence of crown ether exists as solvent-separated ion pair. This observation is consistent with reports of a potassium substituted silyl radical.^16^

### Thermally induced isomerization of 1

**Scheme 2:**
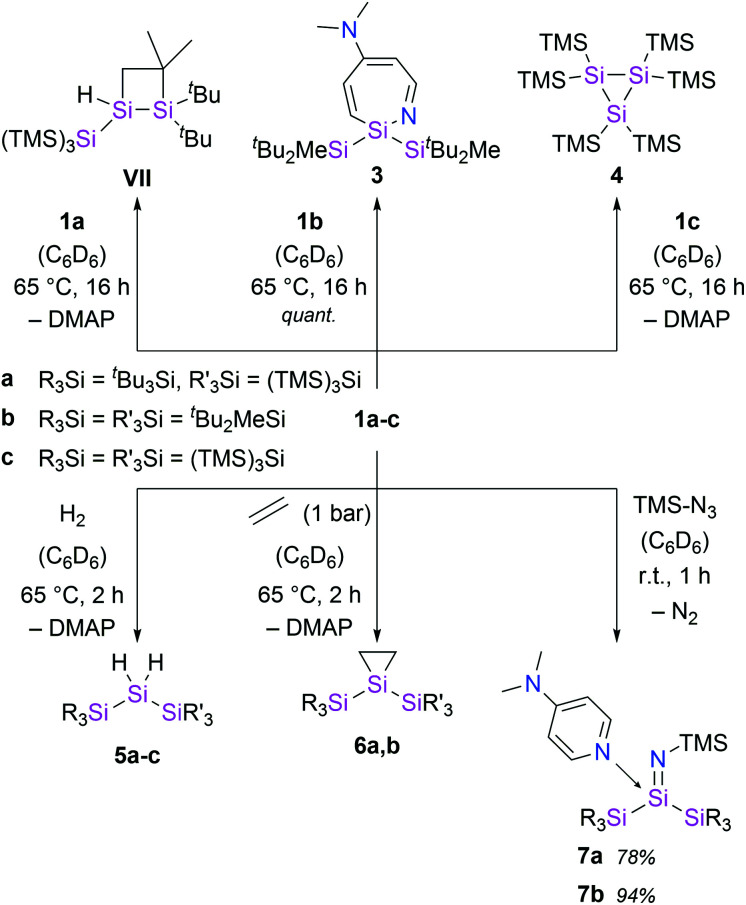
Thermally-induced decomposition of silylenes 1 and synthesis of hydrosilanes 5, siliranes 6 and silaimines 7.

**Fig. 3:**
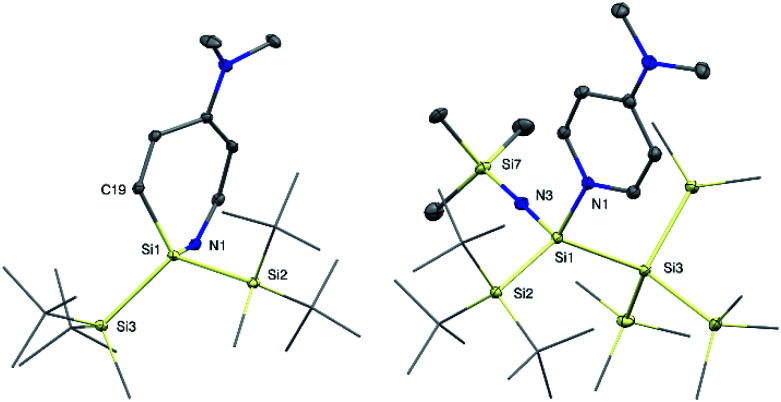
Molecular structures of azasilepin 3 (left) and silaimine 7a (right) with thermal ellipsoids drawn at the 30% probability level. Hydrogen atoms are omitted for clarity. Selected bond lengths [Å] and angles [°]: 3: Si1–N1 1.750(1), Si1–C19 1.878(1), Si1–Si2 2.4144(6), Si2–Si1–Si3 113.74(2), Si2–Si1–N1 109.08(4), N1–Si1–C19 104.71(5); 7a: Si1–Si2 2.453(1), Si1–N1 1.928(2), Si1–N3 1.616(2), N3–Si7 1.660(2), Si2–Si1–Si3 125.08(3), N1–Si1–N3 106.08(8), Si1–N3–Si7 177.1(1).

With the novel silylene complexes 1 in hand, we initially tested their thermal stability. Silylene 1a isomerizes to the respective disiletane VII*via* DMAP dissociation and subsequent C–H bond activation at elevated temperatures (Scheme 2). The same product, that was observed for the decomposition of the donor-free disilene/silylene equilibrium mixture.^5^ Surprisingly, upon heating compound 1b to 65 °C, the silylene fragment inserts into the pyridine ring of DMAP, generating azasilepin 3 by dearomative ring expansion in quantitative yield. Silepin formation *via* insertion of a silylene into an aromatic ring system has previously been reported,^18^ oftentimes either thermally^19^ or photochemically^20^ induced. After transformation from 1b to 3 and thus increase of the coordination number, the ^29^Si NMR signal of the central silicon atom is strongly upfield-shifted to −28.1 ppm. This value is comparable to that of a similar compound, reported by Tokitoh *et al.* from the reaction of a transient, *in situ* generated bis(aryl)silylene with DMAP (−20.8 ppm).^21^ In comparison to 1b, the Si–N bond distance in 3 is shortened by 10% to 1.750(1) Å, indicating a covalent bonding-type instead of the dative interaction in 1b. This bond length is identical to that in Tokitoh's azasilepin.^21^ Furthermore, the Si center adopts a tetrahedral coordination sphere within the boat-shaped, seven-membered heterocyclic ring (*cf.*Fig. 3). In sharp contrast to the related compounds 1a and 1b however, the thermal decomposition of silylene 1c does not proceed *via* C–H, or C–N bond activation, but in fact by silyl migration. At 65 °C, 1c isomerizes under liberation of DMAP to the cyclic silane 4, which was already observed from rearrangement of ((TMS)_3_Si)_2_Si: in the attempted synthesis of the free silylene.^4*a*,*d*^

### Small molecule activation by silylenes 1

Single-site activation of the enthalpically strong, apolar dihydrogen molecule remains a challenging task for low-coordinate silicon compounds. So far, this was only achieved by few acyclic, donor-free silylenes and a masked iminosilyl silylene.^5,18*a*,22^ In fact, to date, there are no reports of H_2_ activation by a silylene base complex.

Although, the thermal decomposition reactions of 1a–c strongly depend on the silyl substituents and proceed *via* three different mechanisms, they are all based on the extreme reactivity of the respective free silylene. Furthermore, the calculated Gibbs free bond-dissociation energy of 1a (15.3 kcal mol^−1^),^5^ which is lower than for the analogous, NHC-coordinated (hypersilyl)(supersilyl)silylene IV^5^ (16.3 kcal mol^−1^)^5^ also suggests a higher reactivity of the DMAP–silylene complexes, compared to the NHC-stabilized bis(silyl)silylenes. Therefore, we conceived compounds 1a–c to be easily accessible synthetic equivalents for these unstable, elusive, donor-free bis(silyl)silylenes and conducted a reactivity study towards activation of small molecules. Indeed, all three DMAP–silylenes underwent dihydrogen addition reactions upon heating to 65 °C, furnishing the reported corresponding dihydrosilanes 5a–c in quantitative yields. (Scheme 2).^5,23^ Remarkably, the oxidative addition of dihydrogen to the DMAP–silylene complexes proceeds in a selective fashion, without the formation of the respective decomposition products. Free DMAP was simply removed from the product by precipitation with one equivalent of SiBr_4_ and subsequent filtration. Notably, no reaction was observed upon exposure of NHC-stabilized bis(silyl)silylenes Ia and IIIa to H_2_, even at elevated temperatures. This result underlines the inherently high reactivity of bis(silyl)silylene–DMAP complexes upon thermal dissociation of the stabilizing donor. Presumably, the H_2_ addition to the silylene fragments of 1 proceeds *via* a bimolecular reaction similar to that proposed for the free silylene (^*t*^Bu_3_Si)((TMS)_3_Si)Si:.^5^

Additional reactivity investigations were carried out with 1a and 1b due to their easier accessibility. Silirane formation – another classical silylene reactivity – was observed after treatment of 1a and 1b with ethylene, yielding compounds 6. The ^29^Si NMR shift of the central Si-atom in 6b (−174.5 ppm) is similar to that of the earlier reported 6a (−164.3 ppm).^5^

Since the isolation of the first silaimine by Wiberg *et al.* in 1985,^24^ a number of these heavier imine analogues have been published. Besides donor free examples,^25^ many silaimines need additional stabilization by a coordinating Lewis base, such as NHCs.^7^ Interestingly, reaction of 1a and 1b with trimethylsilyl azide furnishes the DMAP-coordinated silaimines 7 under liberation of gaseous N_2_. The ^29^Si NMR signals of the central Si atoms in 7a and 7b were observed at −25.9 ppm and −25.5 ppm, respectively. Compared to a silaimine–pyridine adduct (δ^29^Si = −12.6 ppm),^25*b*^ these resonances are slightly upfield-shifted, presumably due to the electropositive silyl groups. In the solid state, compound 7a displays a tetrahedral coordination sphere around the silicon center. Silaimine 7a contains three unique Si–N bonds, distinguishable by their characteristic lengths: a short Si=N bond (1.616(2) Å), a significantly longer Si7–N3 single bond to the TMS group (1.660(2) Å) and an even further elongated, dative Si–N^DMAP^ bond (1.928(2) Å). The central Si=N distance is slightly longer, than in the donor-free silaimines, from the groups of Wiberg and Kira (1.57–1.59 Å)^25*a*,25*c*^ and essentially identical to Klingebiel's silaimine–pyridine adduct (1.611(2) Å).^25*b*^ Interestingly, the geometry of the imino group is almost linear (*θ* = 177.1(1)°). A similar observation was reported by Kira *et al.* and attributed to the electronic properties of the TMS group.^25*c*^ Notably, compound 7a slowly decomposes in solution under liberation of DMAP and probably formation of the donor-free silaimine, which decomposes further to a mixture of unidentified species. Complex 7b instead is stable in solution.

## Conclusions

In summary, we utilized our recently published method to synthesize two novel DMAP-stabilized silylenes 1b and 1c. Compound 1c is the first stable bis(hypersilyl)silylene complex, which could be synthesized so far. Surprisingly, silyl radical 2 was obtained in a related fashion from the over-reduction of the corresponding dibromosilane. The silylene complexes 1a–c turned out to undergo facile oxidative addition with dihydrogen and ethylene at relatively mild conditions. This remarkable reactivity originates from the respective free silylenes, which are generated *in situ* from dissociation of complexes 1. Stabilization of transient bis(silyl)silylenes with DMAP is the only method so far to isolate these species and reactivate their extreme reactivity upon dissociation. Therefore, complexes 1 can be considered easily accessible, stable synthetic equivalents of otherwise elusive bis(silyl)silylenes. Additionally, the unprecedented, DMAP-coordinated silaimines 7 were isolated from the reactions of the silylene complexes with trimethylsilyl azide.

## Conflicts of interest

The authors declare no conflict of interest.

## Supplementary Material

RA-010-C9RA10628F-s001

RA-010-C9RA10628F-s002
