# Supplementary material for: DMAP-stabilized bis(silyl)silylenes as versatile synthons for organosilicon compounds
Source: RSC Adv. 2020 Jan 21;10(6):3402–6. doi: 10.1039/c9ra10628f (PMC9048797; doi:10.1039/c9ra10628f)
Supplement: RA-010-C9RA10628F-s001 [file RA-010-C9RA10628F-s001.pdf]

Electronic Supplementary Material (ESI) for RSC Advances.
This journal is © The Royal Society of Chemistry 2020

Supporting Information

DMAP-stabilized bis(silyl)silylenes as versatile synthons for organosilicon compounds

*Richard Holzner,[†] Dominik Reiter,[†] Philipp Frisch, and Shigeyoshi Inoue**

Department of Chemistry, WACKER-Institute of Silicon Chemistry, Technische Universität München, Lichtenbergstraße 4, 85748 Garching bei München (Germany) *E-mail: s.inoue@tum.de

Table of Contents

1. Experimental Section 3

1.1. General Methods and Instrumentation 3

1.2. ($^{t}Bu_2MeSi)_2Si$:←DMAP (**1b**) 4

1.3. Bis(hypersilyl)silylene DMAP complex (**1c**) 7

1.4. Silyl Radical **2** 9

1.5. Azasilepin **3** 10

1.6. Hexakis(trimethylsilyl)trisilirane (**4**) 14

1.7. Hydrosilanes **5a-c** 16

1.8. Siliranes **6** 17

1.9. Silaimine **7a** 18

1.10. Silaimine **7b** 19

2. X-ray Crystallographic Data 22

2.1. General Information 22

2.2 SC-XRD structures 23

2.3 Crystal data and structural refinement parameters 25

3. References 26

1. Experimental Section

1.1. General Methods and Instrumentation

All manipulations were carried out under exclusion of water and oxygen under an atmosphere of argon 4.6 (≥99.996%) using standard Schlenk and glovebox techniques. The glassware used was heat dried under fine vacuum prior to use. All solvents were refluxed over sodium/benzophenone, freshly distilled under argon and deoxygenated prior to use. PTFE-based grease (*Triboflon III* from *Freudenberg & Co. KG*) was used as sealant. Deuterated benzene (C_6D_6) was obtained from Sigma-Aldrich, dried over Na/K alloy, flask-to-flask condensed, deoxygenated by three freeze-pump-thaw cycles and stored over 3 Å molecular sieves in a glovebox. All NMR samples were prepared under argon in *J. Young* PTFE valve NMR tubes. The NMR spectra were recorded on a *Bruker DRX400* (^{1}H: 400.13 MHz, ^{13}C: 100.62 MHz, ^{29}Si: 79.49 MHz), AV500 (^{1}H: 500.13 MHz) or *AV500C* (^{1}H: 500.36 MHz, ^{13}C: 125.83 MHz, ^{29}Si: 99.41 MHz) spectrometer at ambient temperature (300 K), unless otherwise stated. The ^{1}H, ^{13}C{^{1}H} and ^{29}Si{^{1}H} NMR spectroscopic chemical shifts δ are reported in ppm relative to tetramethylsilane. ^{1}H and ^{13}C{^{1}H} NMR spectra are calibrated against the residual proton and natural abundance carbon resonances of the respective deuterated solvent as internal standard (C_6D_6: δ(^{1}H) = 7.16 ppm and δ(^{13}C) = 128.1 ppm).[S1] The following abbreviations are used to describe signal multiplicities: s = singlet, d = doublet, dd = doublet of doublets, m = multiplet, br = broad. In some NMR spectra, signals from silicone oil (C_6D_6: δ(^{1}H) = 0.29 ppm, δ(^{13}C) = 1.4 ppm and δ(^{29}Si) = -21.8 ppm), originating from the cannulas used (*B. Braun Melsungen AG Sterican®)*, can be observed. EPR spectra were recorded on a *Jeol* jes-Fa200 esr spectrometer with a spectrometer frequency of 9.267 GHz (X-band). Quantitative elemental analyses (EA) were measured with a *EURO EA (HEKAtech)* instrument equipped with a CHNS combustion analyzer at the *Laboratory for Microanalysis* at the *TUM Catalysis Research Center*. Melting Points (m.p.) were determined in sealed glass capillaries under inert gas by a *Büchi M-565* melting point apparatus. Unless otherwise stated, all commercially available chemicals were purchased from *abcr GmbH* or *Sigma-Aldrich* and used without further purification. Hydrogen (H_2) 5.0 (≥99.999%) and ethylene 3.5 (≥99.95%) were purchased from *Westfalen AG* and used as received. The compounds $((TMS)_3Si)_2SiBr_2$,[S2] $(^{t}Bu_2MeSi)_2SiBr_2$,[S3] $(^{t}Bu_3Si)_2SiBr_2$[S4] and $((TMS)_3Si)(^{t}Bu_3Si)Si$←DMAP (**1a**)[S5] were prepared as described in the corresponding references. Potassium graphite (KC_8) was synthesized following a literature reported procedure upon heating a 1:8 mixture of potassium and graphite in a thick-walled, PTFE-capped pressurize-able Schlenk flask to 500 °C until a homogenous bronze powder was obtained.[S6]

1.2. (^{t}Bu$_2$MeSi)$_2$Si:←DMAP (1b)

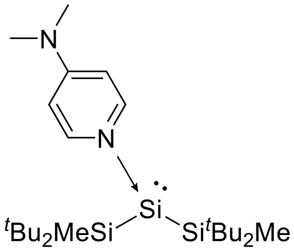

1b

$Si_3C_{25}H_{52}N_2$

464.96 g/mol

THF (10 mL) was added to a mixture of (^{t}Bu$_2$MeSi)$_2$SiBr$_2$ (300 mg, 597 µmol, 1.0 eq.), KC$_8$ (169 mg, 1.25 mmol, 2.1 eq.) and DMAP (72.9 mg, 597 µmol, 1.0 eq.) at ambient temperature. After stirring for 3 hours, the solvent was removed under reduced pressure and the residue was extracted with toluene (3 × 2 mL) to remove KBr and graphite. The solvent was evaporated *in vacuo* and compound **1b** was obtained as red-brown, crystalline solid (233 mg, 551 µmol, 92%). Crystals suitable for SC-XRD analysis were obtained from a cooled (-35 °C) toluene solution of **1b**.

m.p. = 140 °C (decomposition; color change from red-brown to black)

^{1}H NMR (500 MHz, C$_6$D$_6$, 300 K): δ [ppm] = 8.66 (d, ^3J = 7.5 Hz, 2H, *o*-C$^{DMAP}\underline{H}$), 5.42 (d, ^3J = 7.5 Hz, 2H, *m*-C$^{DMAP}\underline{H}$), 1.76 (s, 6H, N(C$\underline{H}_3$)$_2$), 1.41 (s, 36H, C(C$\underline{H}_3$)$_3$), 0.42 (s, 6H, Si(C$\underline{H}_3$)).
^{13}C{^{1}H} NMR (126 MHz, C$_6$D$_6$, 300 K): δ [ppm] = 154.3 (*p*-C^{DMAP}), 153.0 (*o*-C^{DMAP}), 105.2 (*m*-C^{DMAP}), 38.1 (N($\underline{C}$H$_3$)$_2$), 31.2 (C($\underline{C}$H$_3$)$_3$), 22.9 ($\underline{C}$(CH$_3$)$_3$), -3.7 (Si($\underline{C}$H$_3$)).
^{29}Si{^{1}H} NMR (99 MHz, C$_6$D$_6$, 300 K): δ [ppm] = 61.5 ($\underline{Si}$:), 11.8 ($\underline{Si}^t$Bu$_2$Me).

Note: The chemical shifts of DMAP-stabilized silylenes **1** and silaimines **7** are dependent on the concentration of the NMR sample.

**EA:**	$Si_3C_{25}H_{52}N_2$	Calculated [%]:	C (64.58), H (11.27), N (6.03)
		Experimental [%]:	C (64.34), H (11.33), N (5.98)

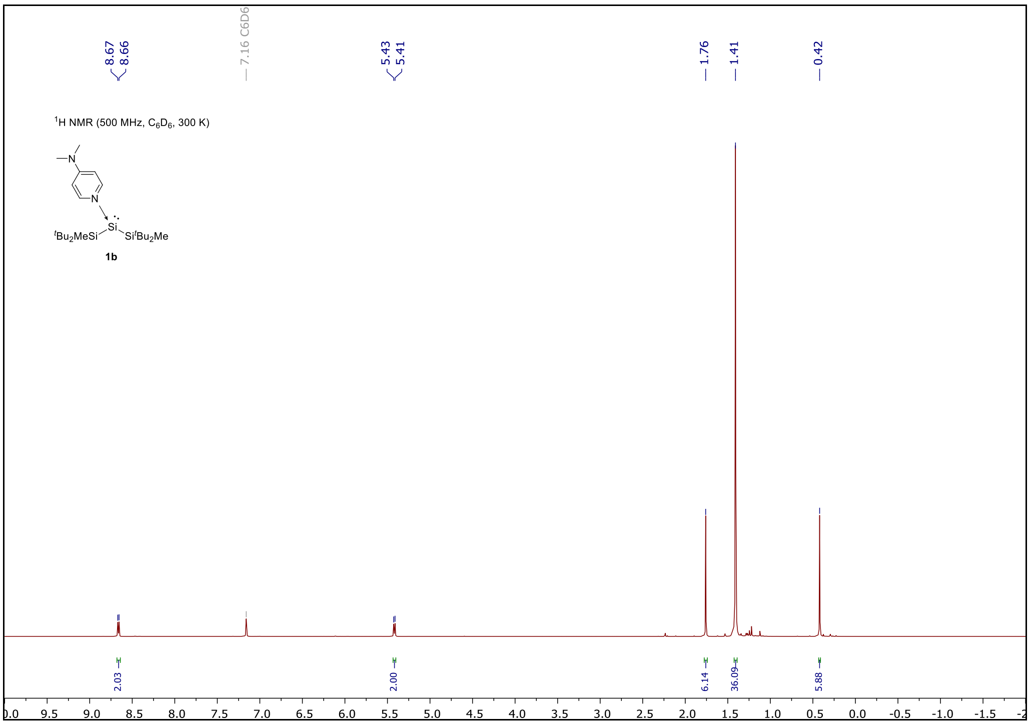

Fig. S1: ^{1}H NMR spectrum (500 MHz) of compound **1b** in C_6D_6 at 300 K.

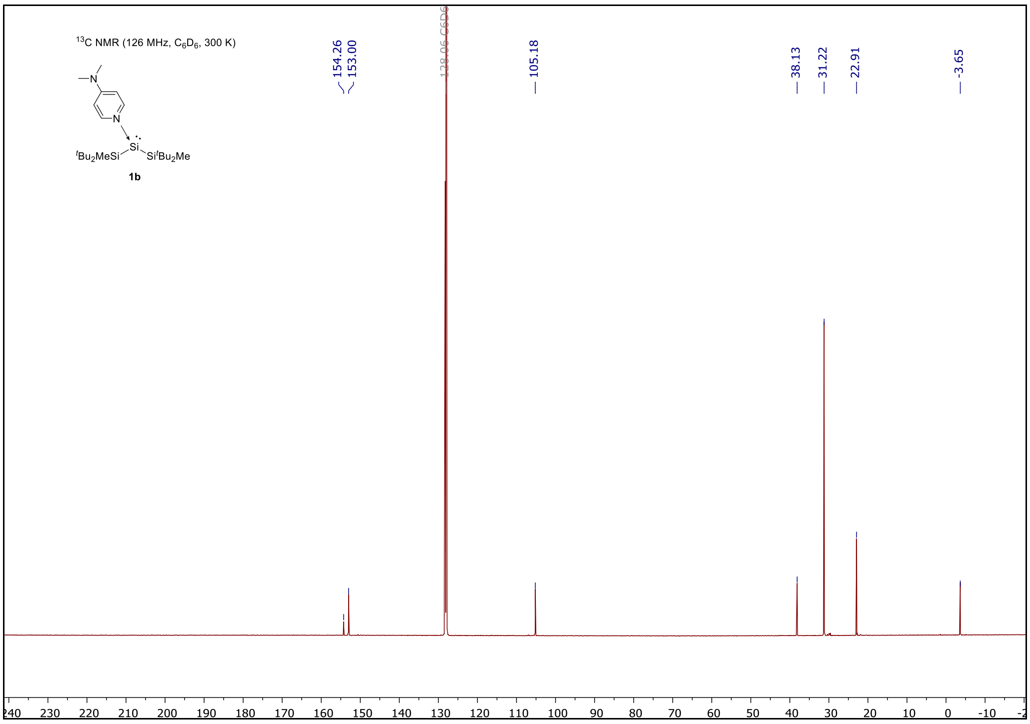

Fig. S2: ^{13}C NMR spectrum (126 MHz) of compound **1b** in C_6D_6 at 300 K.

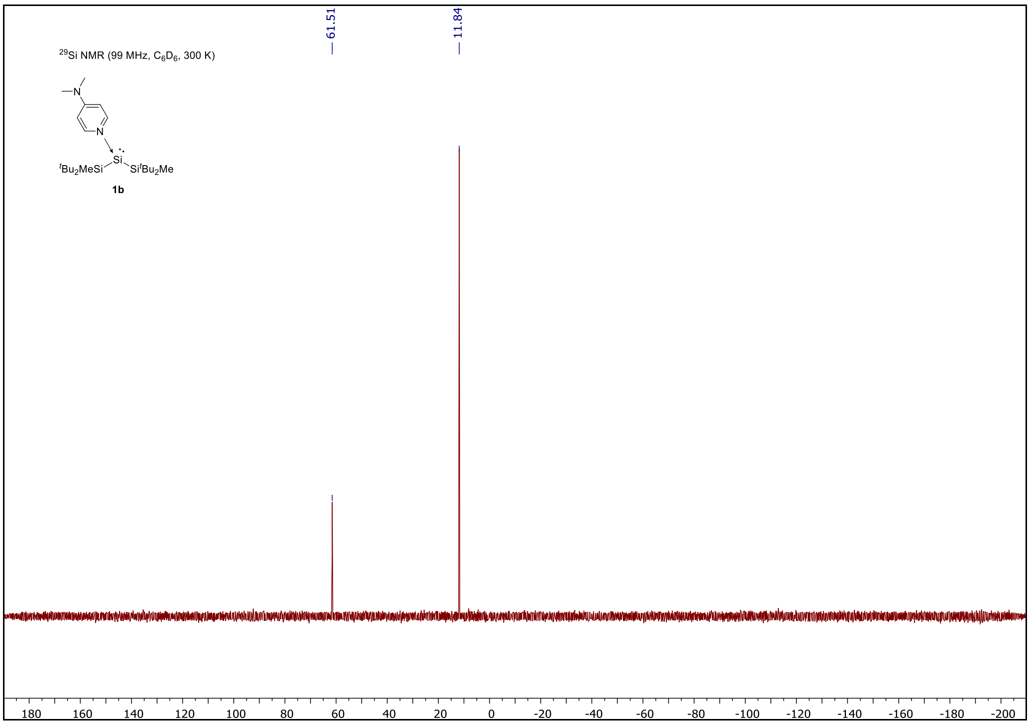

Fig. S3: ^{29}Si NMR spectrum (99 MHz) of compound **1b** in C_6D_6 at 300 K.

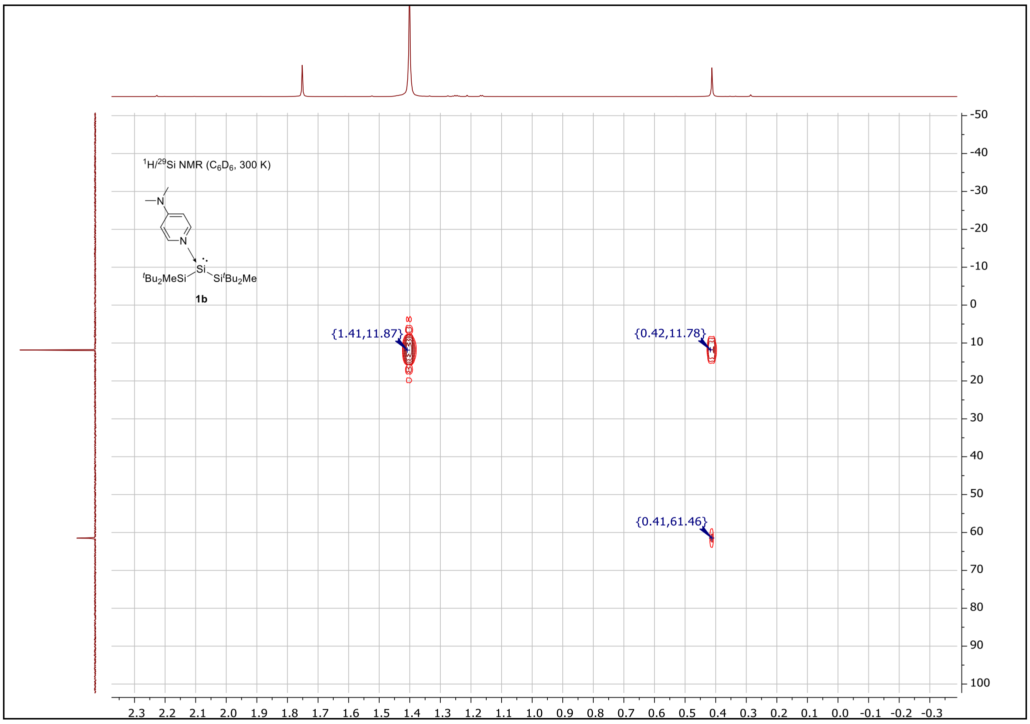

Fig. S4: ^{1}H/^{29}Si HMBC NMR spectrum of compound **1b** in C_6D_6 at 300 K.

1.3. Bis(hypersilyl)silylene DMAP complex (1c)

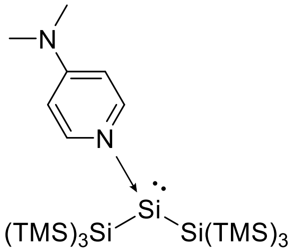

1c

$Si_9C_{25}H_{64}N_2$

645.57 g/mol

THF (10 mL) was added to a mixture of $((TMS)_3Si)_2SiBr_2$ (300 mg, 439 µmol, 1.0 eq.), KC_8 (125 mg, 922 µmol, 2.1 eq.) and DMAP (53.7 mg, 439 µmol, 1.0 eq.) at ambient temperature. After stirring for 3 hours, the solvent was removed under reduced pressure and the residue was extracted with toluene (3 × 2 mL) to remove KBr and graphite. The solvent was evaporated *in vacuo* and compound **1c** was obtained as dark-brown solid.

Note: During the synthesis of **1c**, the concomitant formation of hexakis(trimethylsilyl)trisilirane (**4**) and $Si(TMS)_4$ was observed. Therefore, no sample of **1c** with sufficient purity for elemental analysis was obtained and the yield was not determined.

^{1}H NMR (500 MHz, C_6D_6, 300 K): δ [ppm] = 8.65 (d, ^3J = 7.1 Hz, 2H, *o*-$C^{DMAP}\underline{H}$), 5.67 (d, ^3J = 6.6 Hz, 2H, *m*-$C^{DMAP}\underline{H}$), 1.73 (s, 6H, $N(C\underline{H}_3)_2$), 0.48 (s, 54H, $Si(C\underline{H}_3)_3$).
^{29}Si{^{1}H}(NMR (99 MHz, C_6D_6, 300 K): δ [ppm] = 72.5 (*Si*:), -9.7 (<u>*Si*</u>$(CH_3)_3$), -121.8 (<u>*Si*</u>$(TMS)_3$).

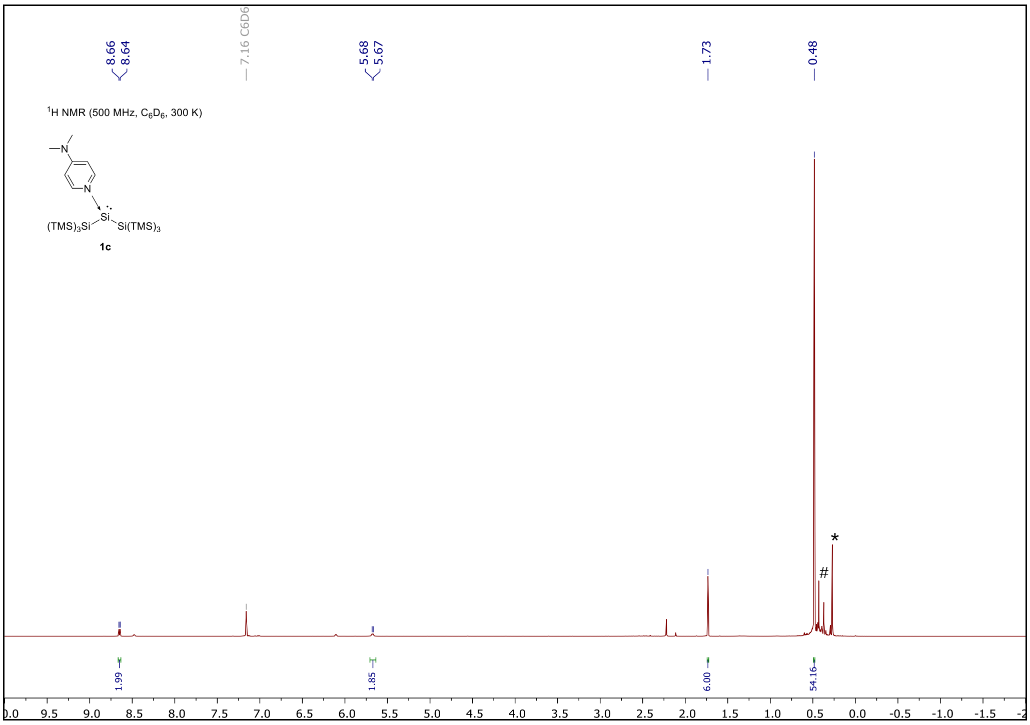

Fig. S5: ^{1}H NMR spectrum (500 MHz) of compound **1c** in C_6D_6 at 300 K. Signals labeled with * and # belong to $Si(TMS)_4$ and hexakis(trimethylsilyl)trisilirane (**4**), respectively.

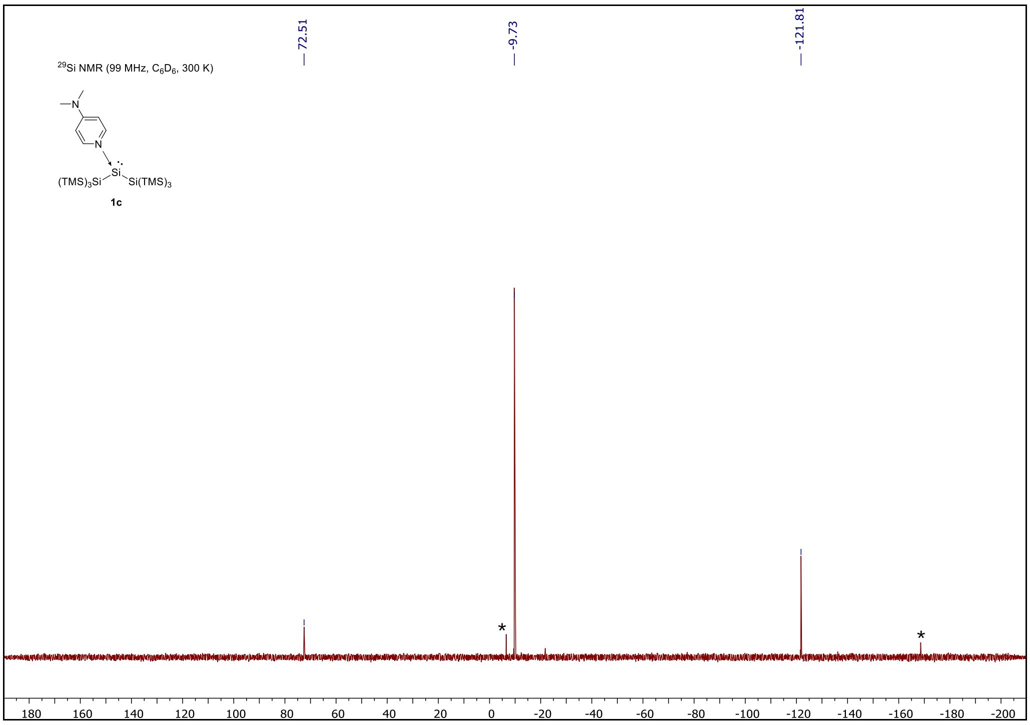

Fig. S6: ^{29}Si NMR spectrum (99 MHz) of compound **1c** in C_6D_6 at 300 K. Signals labeled with * belong to hexakis(trimethylsilyl)trisilirane (**4**).

1.4. Silyl Radical 2

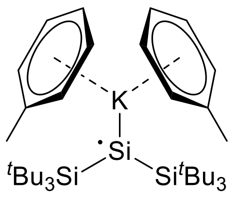

2

$Si_3C_{38}H_{70}K$

650.33 g/mol

Precooled THF (10 mL, -78 °C) was added to a mixture of $(^tBu_3Si)_2SiBr_2$ (100 mg, 170 µmol, 1.0 eq.) and KC_8 (92.2 mg, 682 µmol, 3.5 eq.). The reaction mixture was allowed to warm to room temperature over 16 hours and the solvent was subsequently removed under reduced pressure. Concomitantly formed KBr and graphite were separated by extracting the residue with toluene (3 × 4 mL). Evaporation of the solvent *in vacuo* and subsequent washing of the residue with *n*-hexane (3 × 2 mL) afforded compound **2** as orange-brown solid (54.1 mg, 88.5 µmol, 52%). Crystals suitable for SC-XRD analysis were obtained from a cooled (-35 °C) solution of **2** in toluene.

m.p. = 60 °C (decomposition; color change to dark red)

EPR (toluene, 286 K) g = 2.0056, $a(\alpha\text{-}^{29}Si)$ = 2.92 mT

Note: Compound **2** is completely NMR silent. Elemental analysis was not matching, presumably because an unquantifiable amount of coordinating toluene was removed during drying compound **2** in fine vacuum. Due to its extreme air and moisture sensitivity and the fact, that it is not stable in toluene, no satisfactory spectroscopic data of **2** was obtained before addition of crown ether (18-C-6). With crown ether however, one signal in the EPR spectrum was observed (Fig. **S7**). Hyperfine coupling with the $\beta\text{-}^{29}Si$ nuclei was not visible.

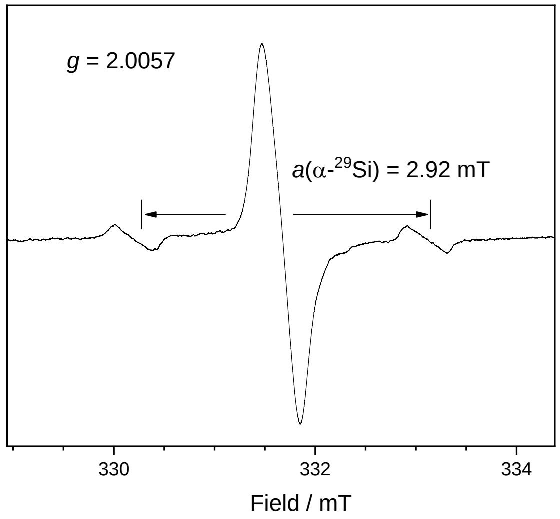

Fig. S7: X-band EPR spectrum of compound **2** + crown ether (18-C-6) in toluene (1×10^{-4} M, 286 K).

1.5. Azasilepin 3

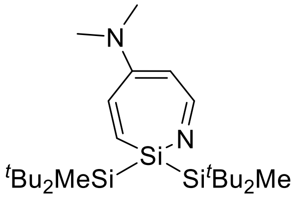

3

$Si_3C_{25}H_{52}N_2$

464.96 g/mol

A solution of DMAP-stabilized silylene **1b** (36.0 mg, 85.1 µmol) in benzene (2 mL) was heated to 65 °C for 16 h. The color changed from deep-brown to yellow. Evaporation of the solvent, afforded compound **3** as yellow solid (36.0 mg, 85.1 µmol, quant.). Crystals suitable for SC-XRD analysis were obtained from a cooled (-35 °C) solution of **3** in *n*-hexane.

^{1}H NMR (500 MHz, C_6D_6, 300 K): δ [ppm] = 8.34 (d, ^3J = 4.7 Hz, 1H, NC*H*), 6.27 (dd, ^3J = 15.5 Hz, ^4J = 2.5 Hz, 1H, SiCHC*H*), 6.01 (d, ^3J = 15.5 Hz, 1H, SiC*H*), 4.68 (dd, ^3J = 4.7 Hz, ^4J = 2.5 Hz, 1H, NCHC*H*), 2.27 (s, 6H, N(C*H*$_3$)$_2$), 1.29 (s, 18H, C(C*H*$_3$)$_3$), 1.19 (s, 18H, C(C*H*$_3$)$_3$), 0.37 (s, 6H, Si(C*H*$_3$)).

^{13}C{^{1}H} NMR (126 MHz, C_6D_6, 300 K): δ [ppm] = 165.4 (SiN*C*H), 156.2 (*C*NMe$_2$), 138.2 (NCH*C*H), 137.4 (Si*C*HCH), 105.8 (SiCH*C*H), 40.3 (N(*C*H$_3$)$_2$), 30.4 (Si(C(*C*H$_3$)$_3$), 30.1 (Si(C(*C*H$_3$)$_3$), 22.5 (Si(*C*(CH$_3$)$_3$), 21.6 (Si(*C*(CH$_3$)$_3$), -4.9 (Si(*C*H$_3$)).

^{29}Si{^{1}H} NMR (99 MHz, C_6D_6, 300 K): δ [ppm] = 2.0 (*Si*^{t}Bu$_2$Me), -28.1 (*Si*N).

**EA:**	$Si_3C_{25}H_{52}N_2$	Calculated [%]:	C (64.58), H (11.27), N (6.03)
		Experimental [%]:	C (64.62), H (11.47), N (5.99)

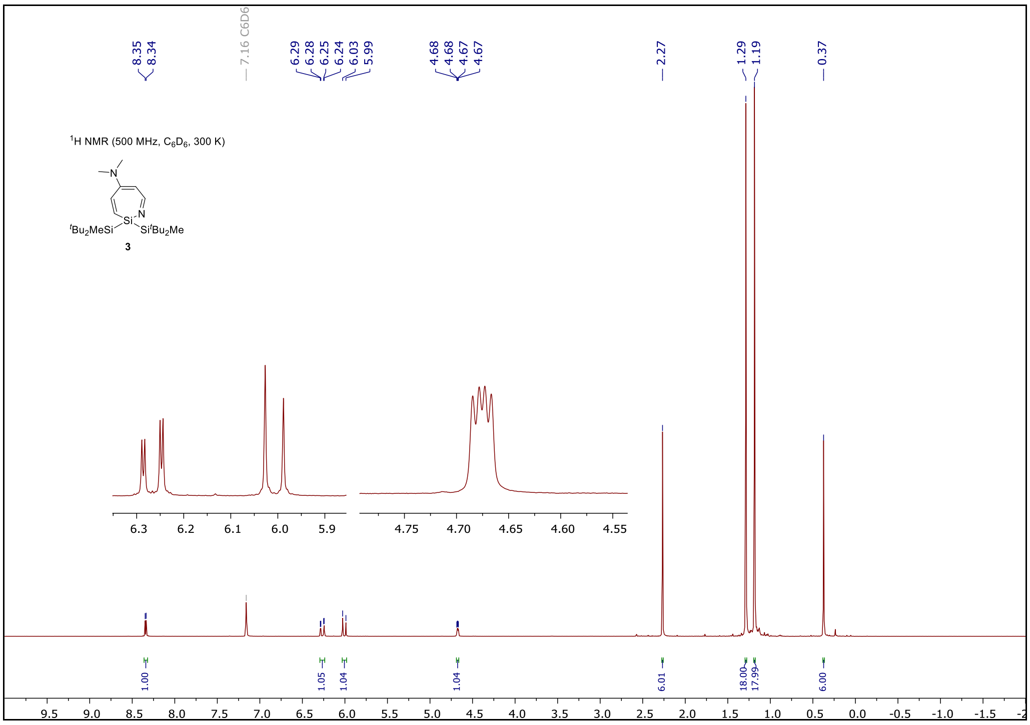

Fig. S8: ^{1}H NMR spectrum (500 MHz) of compound **3** in C_6D_6 at 300 K.

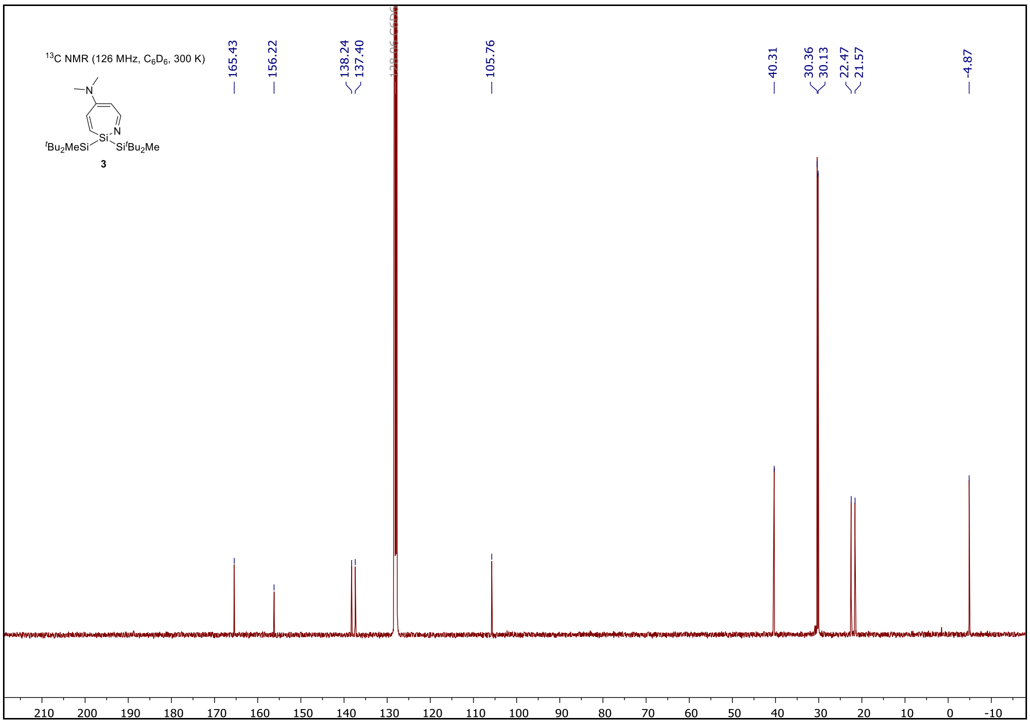

Fig. S9: ^{13}C NMR spectrum (126 MHz) of compound **3** in C_6D_6 at 300 K.

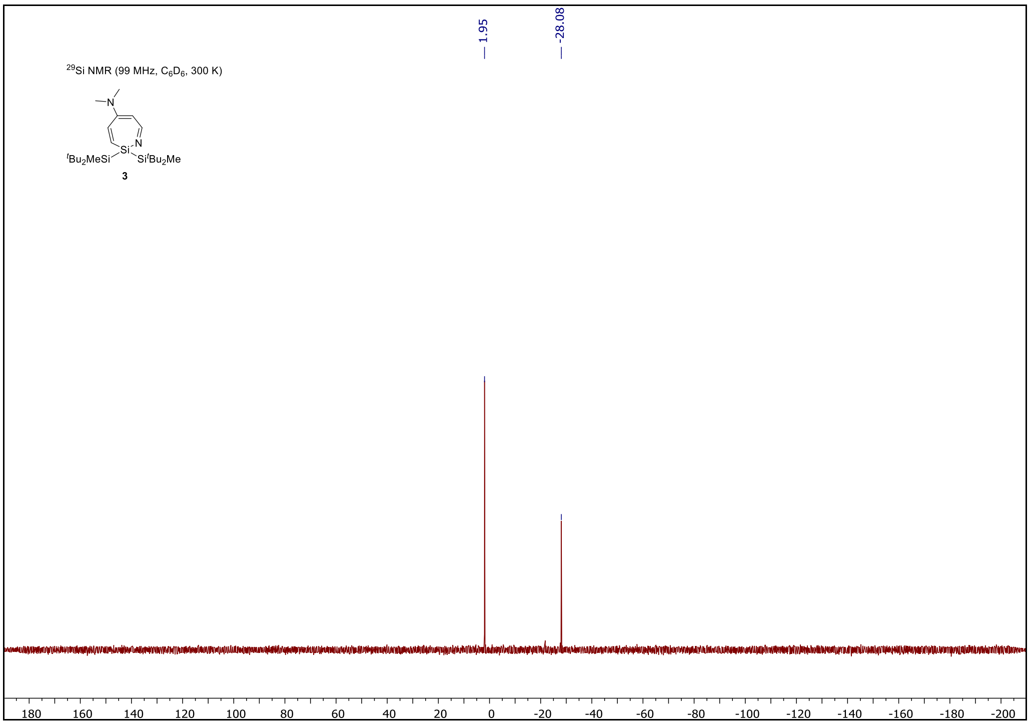

Fig. S10: ^{29}Si NMR spectrum (99 MHz) of compound **3** in C_6D_6 at 300 K.

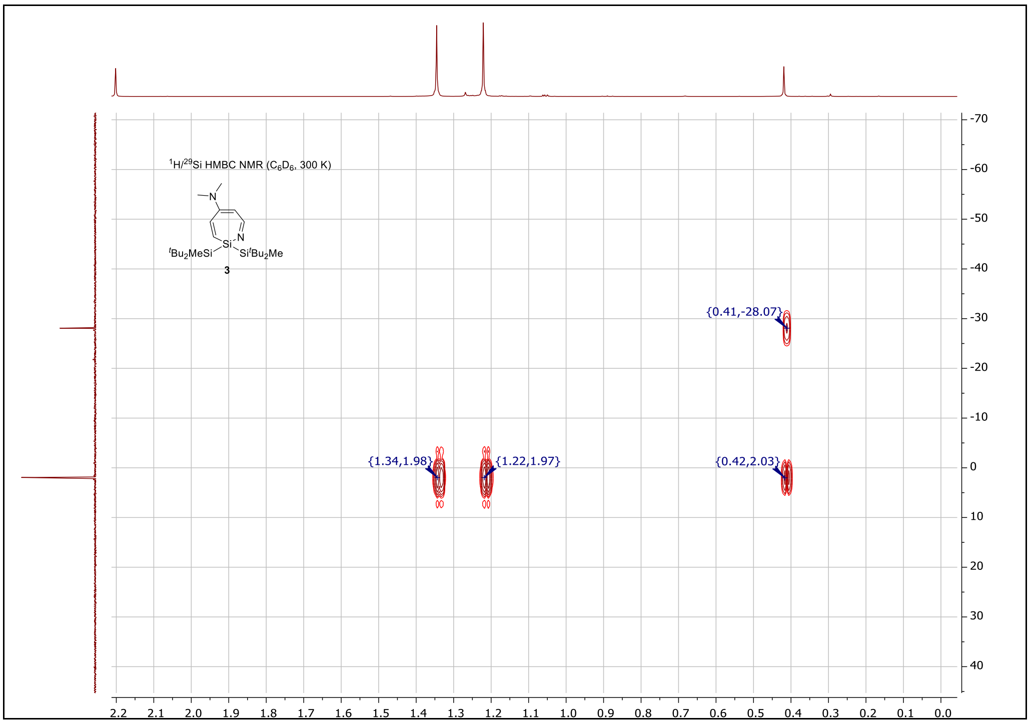

Fig. S11: ^{1}H/^{29}Si HMBC NMR spectrum of compound **3** in C_6D_6 at 300 K.

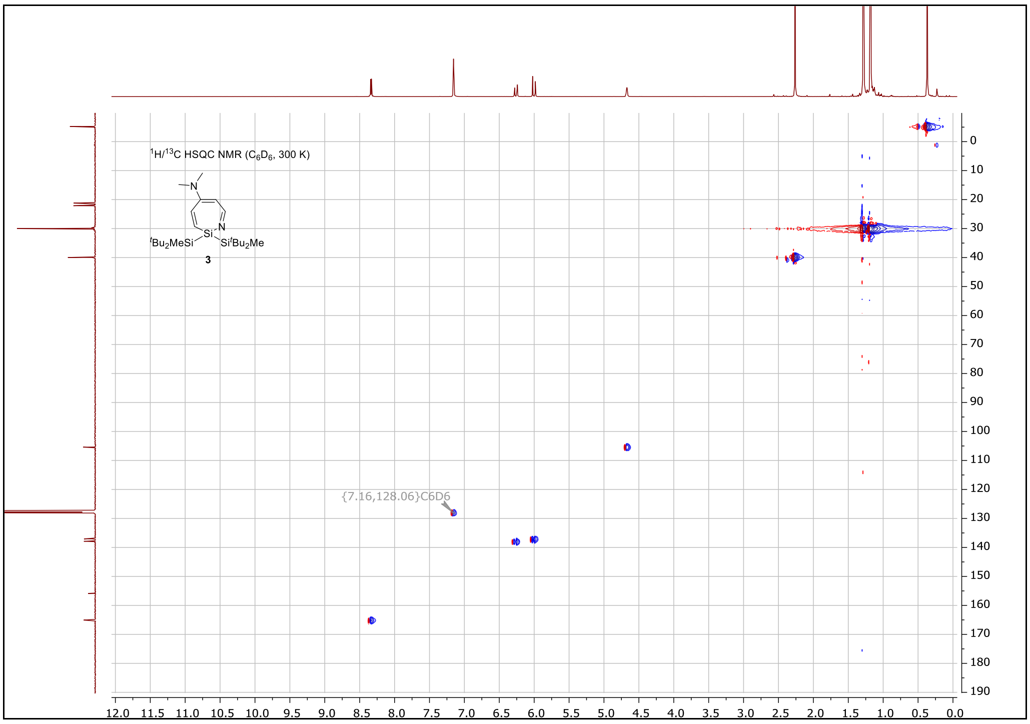

Fig. S12: $^{1}H/^{13}C$ HSQC NMR spectrum of compound **3** in C_6D_6 at 300 K.

1.6. Hexakis(trimethylsilyl)trisilirane (4)

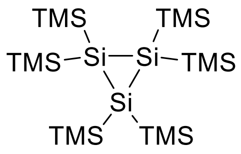

4

$Si_9C_{18}H_{54}$

523.40 g/mol

A toluene solution of crude silylene **1c** (50 mg) was heated to 65 °C for 16 hours. After cooling down to ambient temperature, liberated DMAP was separated by precipitation with $SiBr_4$ (26.9 mg, 77.5 µmol, 1.0 eq.) and subsequent filtration. The resulting solution was concentrated under reduced pressure and compound **4** was obtained by crystallization at -35 °C as colorless solid (30.1 mg).

Note: Compound **4** has already been reported by Klinkhammer *et al.* from the attempted synthesis of the free silylene $((TMS)_3Si)_2Si$:.[S7] Therefore, we did not analyze it further. DMAP forms an unidentified adduct with $SiBr_4$ which is insoluble in common organic solvents. Thus, this adduct was not further analyzed.

^{1}H NMR (500 MHz, C_6D_6, 300 K): δ [ppm] = 0.43 (s, 54H, Si(C<u>*H*</u>$_3$)$_3$).
^{13}C{^{1}H} NMR (126 MHz, C_6D_6, 300 K): δ [ppm] = 4.7 (Si(<u>*C*</u>H$_3$)$_3$).
^{29}Si{^{1}H} NMR (99 MHz, C_6D_6, 300 K): δ [ppm] = -6.5 (<u>*Si*</u>(CH$_3$)$_3$), -168.6 (<u>*Si*</u>(TMS)$_2$).

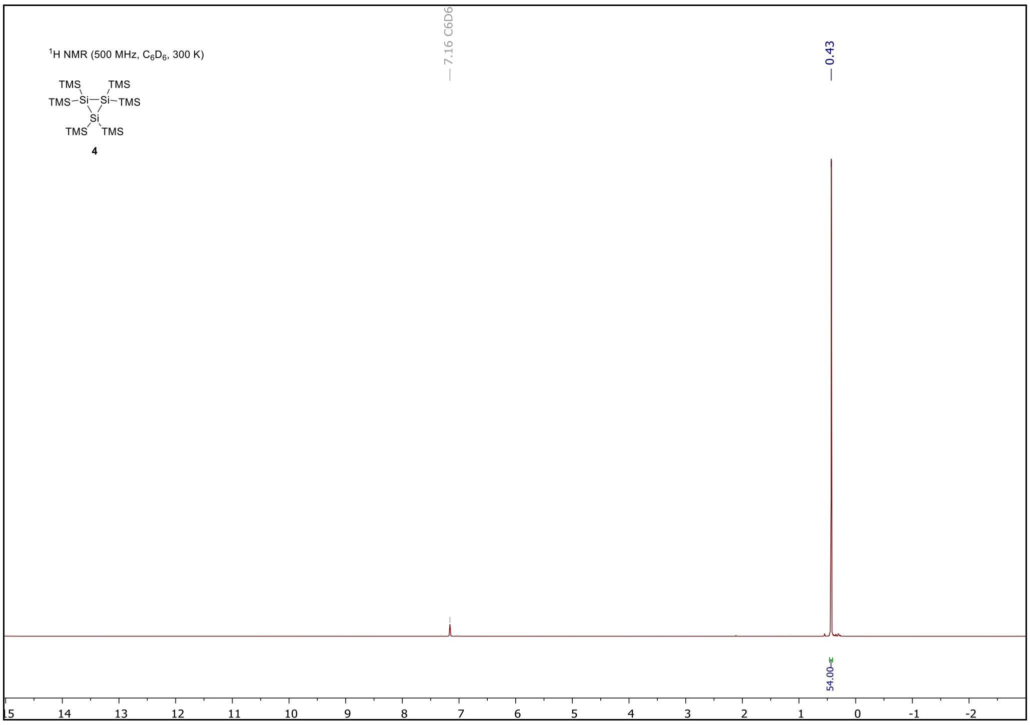

Fig. S13: ^{1}H NMR spectrum (500 MHz) of compound **4** in C_6D_6 at 300 K.

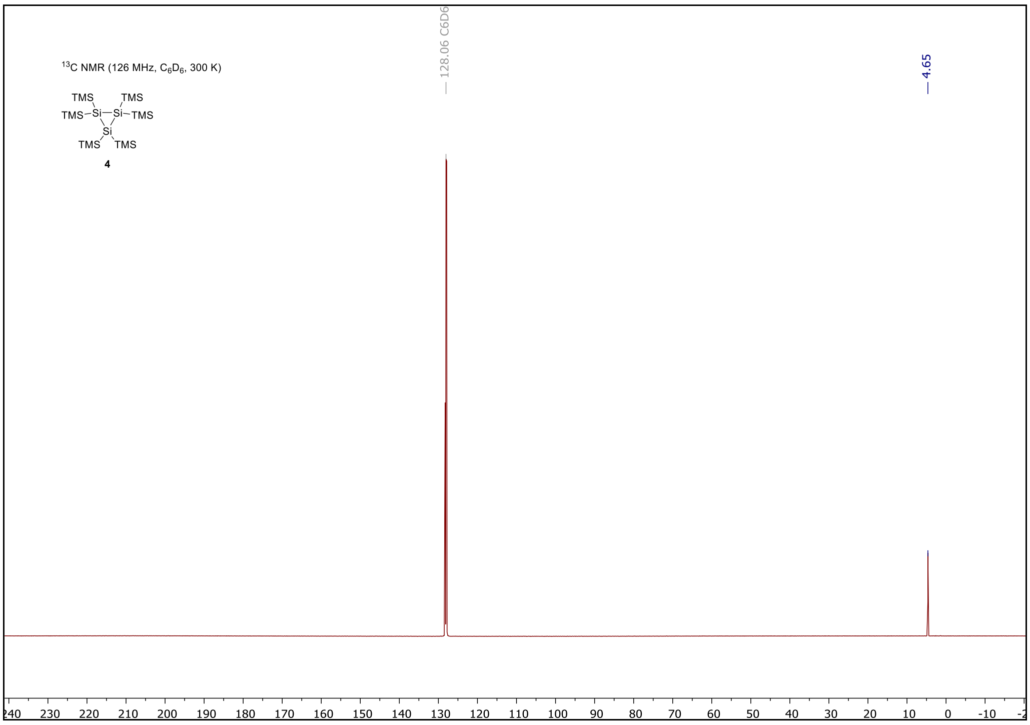

Fig. S14: ^{13}C NMR spectrum (126 MHz) of compound **4** in C_6D_6 at 300 K.

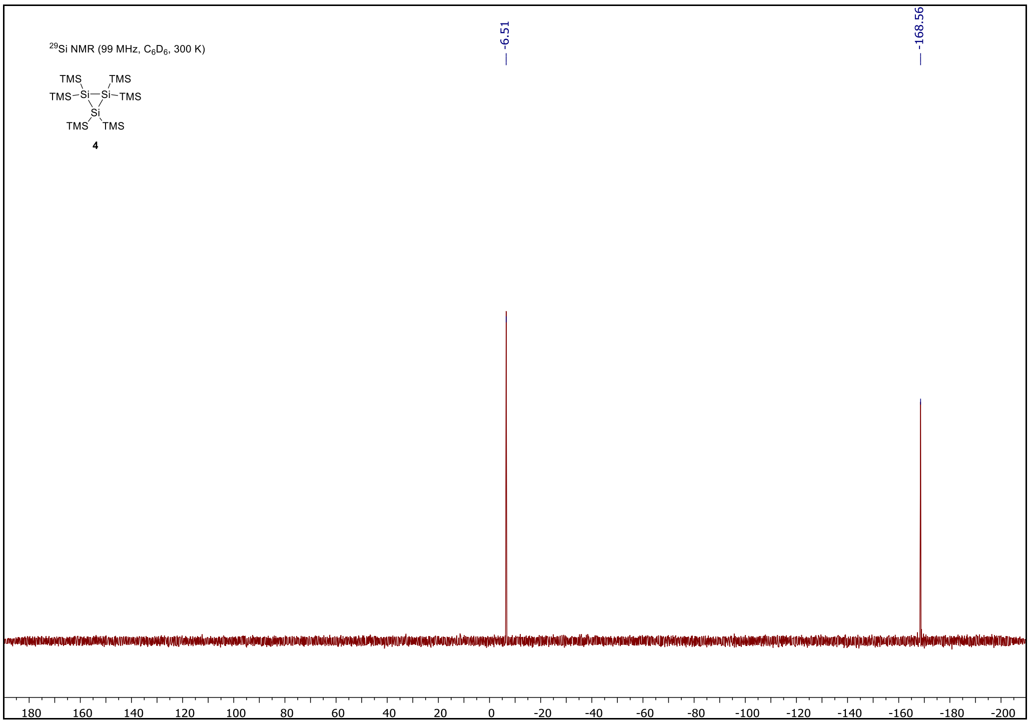

Fig. S15: ^{29}Si NMR spectrum (99 MHz) of compound **4** in C_6D_6 at 300 K.

1.7. Hydrosilanes 5a-c

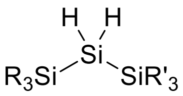

5a R = $Si^{t}Bu_3$, R' = $Si(TMS)_3$
5b R = R' = $Si(TMS)_3$
5c R = R' = $Si^{t}Bu_2Me$

In a pressurizable Schlenk flask, a solution of the respective DMAP-stabilized silylenes **1a-c** (100 µmol, 1.0 eq.) in toluene (5 mL) was frozen in liquid nitrogen, degassed and exposed to dihydrogen (1 bar). The reaction mixture was subsequently heated to 65 °C for 2 hours. Decolorization indicated full conversion. Concomitantly formed free DMAP was separated by precipitation with $SiBr_4$ (100 µmol, 1.0 eq.) and filtration. The solvent was removed under reduced pressure to afford hydrosilanes **5** as colorless solids in quantitative yields. Compounds **5a-c** were identified by comparison of NMR spectral data with corresponding literature reports (**5a**,[S5] **5b**,[S2] **5c**[S3]).

1.8. Siliranes 6

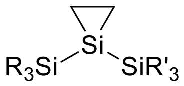

6a R = Si^{t}Bu$_3$, R' = Si(TMS)$_3$
6b R = R' = Si^{t}Bu$_2$Me

The synthesis of siliranes **6** was conducted by a similar procedure than than that for hydrosilanes **5** (*vide supra*). Instead of H_2, the DMAP-silylene complexes **1a** and **1b** were exposed to ethylene (1 bar). The compounds **6** were obtained as colorless solids. Silirane **6a** was identified by comparison of NMR spectral data with literature reports.[S5] Compound **6b** was identified by multinuclear NMR spectroscopy.

6b

^{1}H NMR (500 MHz, C_6D_6, 300 K): δ [ppm] = 1.10 (s, 36H, C(C$\underline{H}_3$)$_3$), 0.81 (s, 4H, C$\underline{H}_2$), 0.10 (s, 6H, Si(C$\underline{H}_3$)).
^{13}C NMR (126 MHz, C_6D_6, 300 K): δ [ppm] = 29.9 (C($\underline{C}$H$_3$)$_3$), 21.8 ($\underline{C}$(CH$_3$)$_3$), -0.7 ($\underline{C}$H$_2$), -6.3 (Si$\underline{C}$H$_3$)
^{29}Si{^{1}H} NMR (99 MHz, C_6D_6, 300 K): δ [ppm] = 11.2 ($\underline{Si}$^{t}Bu$_2$Me), -174.5 ($\underline{Si}$CH$_2$).

1.9. Silaimine 7a

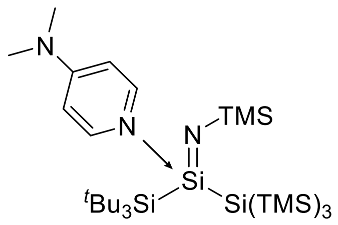

7a

$Si_7C_{31}H_{73}N_3$

684.54 g/mol

Trimethylsilyl azide (11.6 mg, 100 µmol, 1.0 eq.) was added to a solution of silylene **1a** (60.0 mg, 100 µmol, 1.0 eq.) in benzene (2 mL) at ambient temperature. Decolorization from dark-brown to yellow and concomitant N_2 evolution was observed. After stirring the mixture for 1 hour, evaporation of the solvent under reduced pressure afforded compound **7a** as yellow solid (53.8 mg, 78.6 µmol, 78%). Crystals suitable for SC-XRD analysis were obtained from a cooled (-35 °C) *n*-hexane solution of **7a**.

Note: Compound **7a** decomposes is solution under liberation of DMAP. Presumably, the donor-free silaimine is formed that decomposes further to an unidentified mixture of products.

^{1}H NMR (500 MHz, C_6D_6, 300 K): δ [ppm] = 9.42 (br. s, 1H, *o*-C^{DMAP}*H*), 8.50 (br. s, 1H, *o*-C^{DMAP}*H*), 6.06 (d, ^3J = 6.9 Hz, 2H, *m*-C^{DMAP}H), 1.83 (s, 6H, N(C*H*$_3$)$_2$), 1.41 (s, 27H, Si(C(C*H*$_3$)$_3$)), 0.69 (s, 9H, NSi(C*H*$_3$)$_3$), 0.47 (s, 27H, Si(Si(C*H*$_3$)$_3$)$_3$).

^{13}C NMR (126 MHz, C_6D_6, 300 K): δ [ppm] = 155.9 (*p*-C^{DMAP}), 150.6 (*o*-C^{DMAP}), 106.9 (*m*-C^{DMAP}), 38.3 (N(*C*H$_3$)$_2$), 33.3 (C(*C*H$_3$)$_3$), 25.1 (*C*(CH$_3$)$_3$), 7.7 (NSi(*C*H$_3$), 5.3 (Si(Si(*C*H$_3$)$_3$).

^{29}Si{^{1}H} NMR (99 MHz, C_6D_6, 300 K): δ [ppm] = 2.0 (*Si*^{t}Bu$_3$), -9.4 (Si(*Si*Me$_3$)$_3$), -25.1 (N(*Si*Me$_3$), -25.9 (*Si*=N), -121.3 (*Si*(SiMe$_3$)$_3$).

1.10. Silaimine 7b

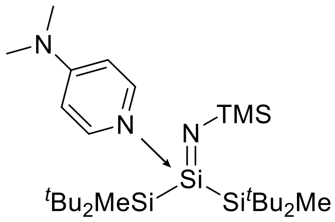

7b

$Si_4C_{28}H_{61}N_3$

552.16 g/mol

Analog to the synthesis of **7a** (*vide supra*) silylene **1b** (25 mg, 53.8 µmol, 1.0 eq.) was treated with trimethylsilyl azide (6.19 mg, 53.8 µmol, 1.0 eq.) to afford silaimine **7b** as yellow, crystalline solid (28.4 mg, 51.4 mmol, 96%).

^{1}H NMR (500 MHz, C_6D_6, 300 K): δ [ppm] = 8.82-8.81 (m, 2H, *o*-C^{DMAP}<u>*H*</u>), 5.79 (d, ^3J = 7.3 Hz, 2H, *m*-C^{DMAP}<u>*H*</u>), 1.80 (s, 6H, N(C<u>*H*</u>$_3$)$_2$), 1.34 (s, 18H, C(C<u>*H*</u>$_3$)$_3$), 1.16 (s, 18H, C(C<u>*H*</u>$_3$)$_3$), 0.72 (s, 9H, Si(CH$_3$)$_3$), 0.42 (s, 6H, Si(C<u>*H*</u>$_3$)).

^{13}C{^{1}H} NMR (126 MHz, C_6D_6, 300 K): δ [ppm] = 155.8 (*p*-C^{DMAP}), 145.7 (*o*-C^{DMAP}), 106.3 (*m*-C^{DMAP}), 38.3 (N(<u>*C*</u>H$_3$)$_2$), 31.1 (C(<u>*C*</u>H$_3$)$_3$), 31.1 (C(<u>*C*</u>H$_3$)$_3$), 22.5 (<u>*C*</u>(CH$_3$)$_3$), 22.3 (<u>*C*</u>(CH$_3$)$_3$), -4.5 (Si(<u>*C*</u>H$_3$)).

^{29}Si{^{1}H} NMR (99 MHz, C_6D_6, 300 K): δ [ppm] = -8.8 (<u>*Si*</u>^{t}Bu$_2$Me), -24.9 (<u>*Si*</u>Me$_3$), -25.5 (<u>*Si*</u>=N).

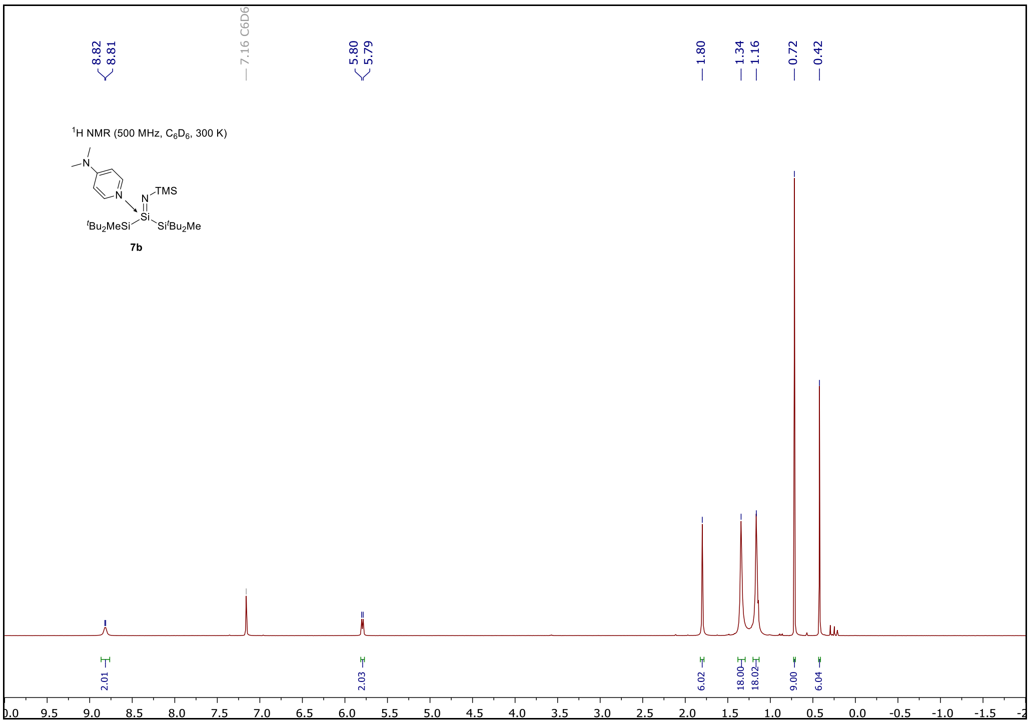

Fig. S16: ^{1}H NMR spectrum (500 MHz) of compound **7b** in C_6D_6 at 300 K.

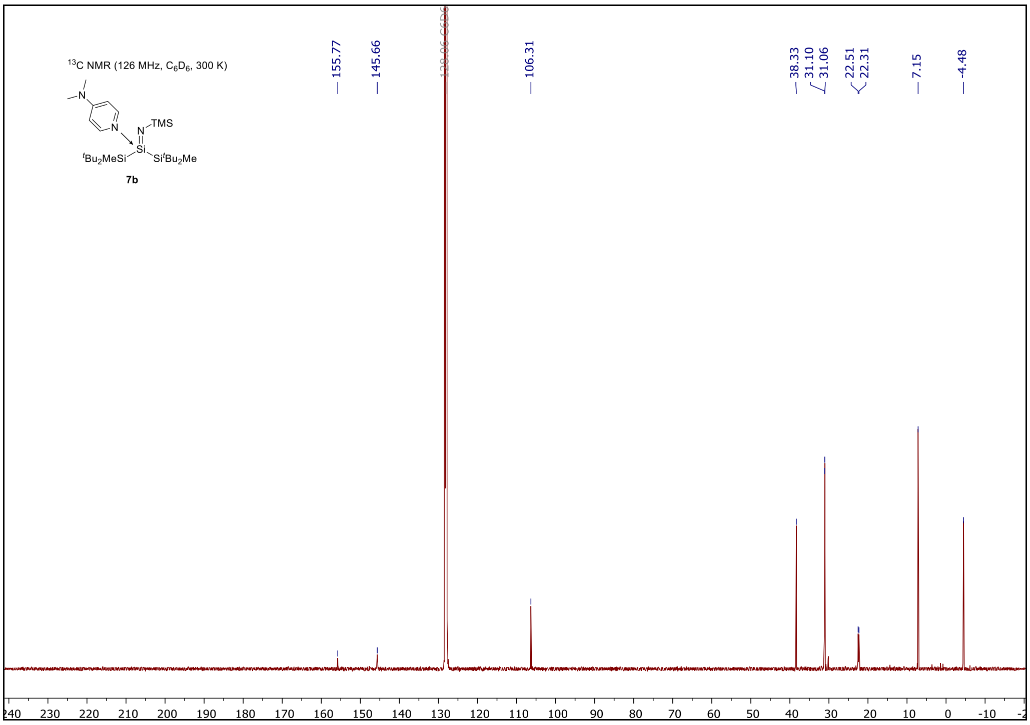

Fig. S17: ^{13}C NMR spectrum (126 MHz) of compound **7b** in C_6D_6 at 300 K.

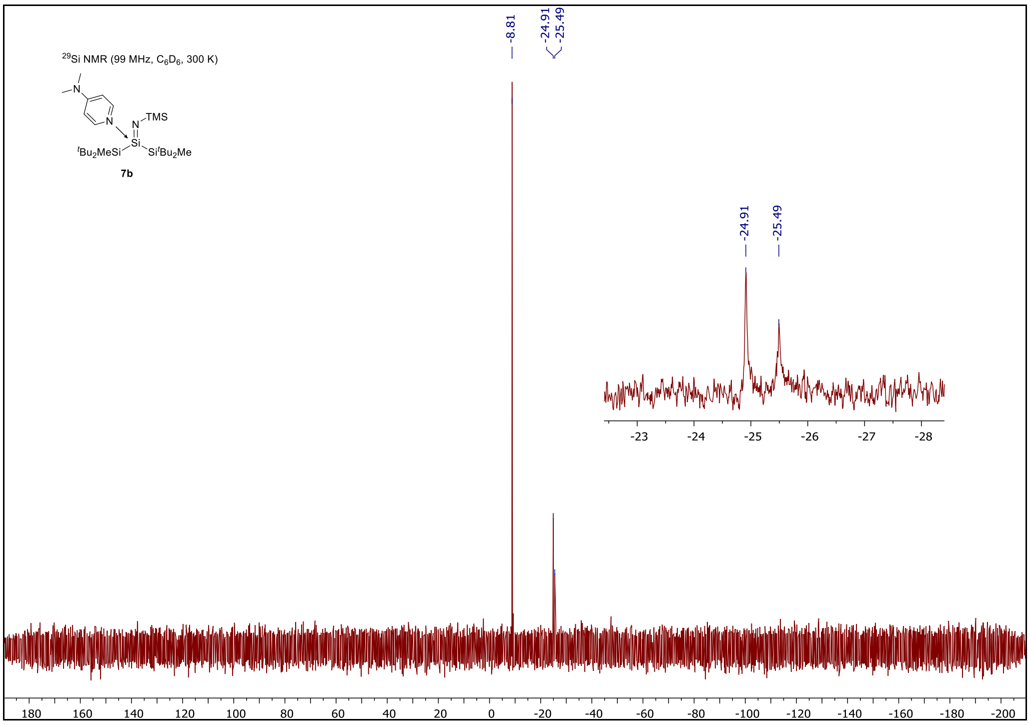

Fig. S18: ^{29}Si NMR spectrum (99 MHz) of compound **7b** in C_6D_6 at 300 K.

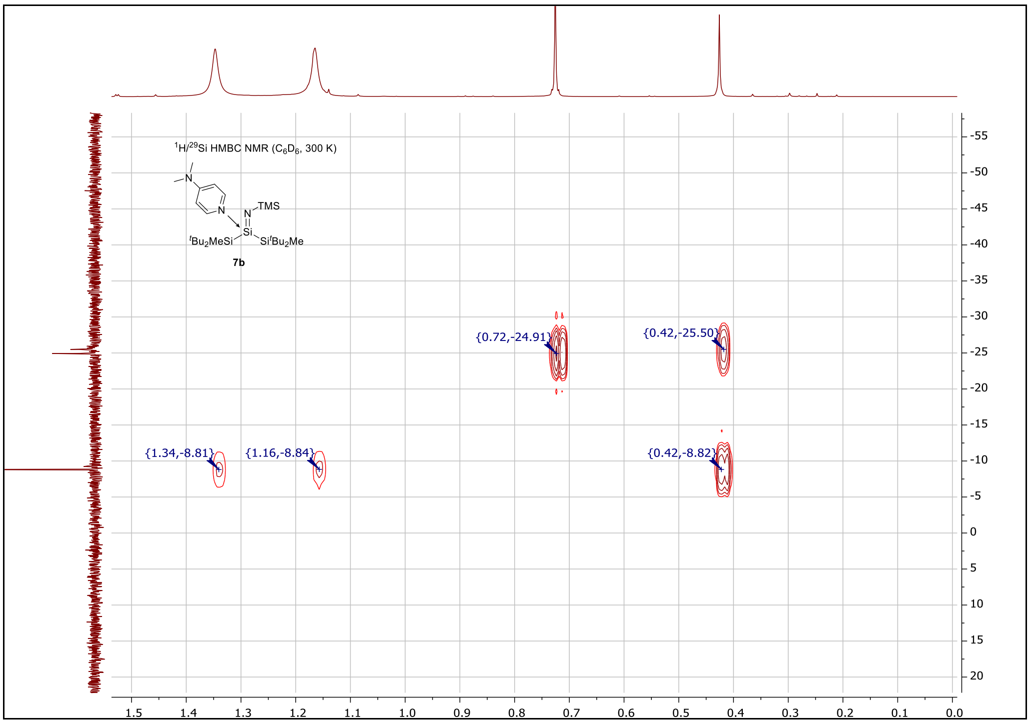

Fig. S19: $^1H/^{29}Si$ HMBC NMR spectrum of compound **7b** in C_6D_6 at 300 K.

2. X-ray Crystallographic Data

2.1. General Information

The X-ray intensity data of **2** were collected on an X-ray single crystal diffractometer equipped with a CMOS detector (*Bruker Photon-100*), a rotating anode (*Bruker TXS*) with MoKα radiation (λ = 0.71073 Å) and a *Helios* mirror optic by using the *APEX III* software package.[S8] The X-ray intensity data of **1b** and **7a** were collected on an X-ray single crystal diffractometer equipped with a CMOS detector (*Bruker Photon-100*), an IMS microsource with MoKα radiation (λ = 0.71073 Å) and a *Helios* mirror optic by using the *APEX III* software package.[S8] The X-ray intensity data of **3** was collected on an X-ray single crystal diffractometer equipped with a CCD detector (*Apex II CCD*), a fine-focus sealed tube with MoKα radiation (λ = 0.71073 Å) and a *Triumph* monochromator by using the *APEX II/III* software package.[S8] The measurements were performed on single crystals coated with the perfluorinated ether *Fomblin® Y*. The crystal was fixed on the top of a micro sampler, transferred to the diffractometer and frozen under a stream of cold nitrogen. A matrix scan was used to determine the initial lattice parameters. Reflections were merged and corrected for Lorenz and polarization effects, scan speed, and background using *SAINT*.[S9] Absorption corrections, including odd and even ordered spherical harmonics were performed using *SADABS*.[S9] Space group assignments were based upon systematic absences, E statistics, and successful refinement of the structures. Structures were solved by direct methods with the aid of successive difference Fourier maps, and were refined against all data using the *APEX III* software in conjunction with *SHELXL-2014*[S10] and *SHELXLE*.[S11] All H atoms were placed in calculated positions and refined using a riding model, with methylene and aromatic C–H distances of 0.99 and 0.95 Å, respectively, and $U_{iso}(H) = 1.2 \cdot U_{eq}(C)$. Full-matrix least-squares refinements were carried out by minimizing $\Delta w(F_o^2 - F_c^2)$[S9] with *SHELXL-97* weighting scheme.[S12] Neutral atom scattering factors for all atoms and anomalous dispersion corrections for the non-hydrogen atoms were taken from International Tables for Crystallography.[S13] The images of the crystal structures were generated by *Mercury*.[S14] The CCDC numbers CCDC-1967942 (**1b**), CCDC-1967943 (**2**), CCDC-1967944 (**3**) and CCDC-1967945 (**7a**) contain the supplementary crystallographic data for the structures. These data can be obtained free of charge from the Cambridge Crystallographic Data Centre *via* https://www.ccdc.cam.ac.uk/structures/.

2.2 SC-XRD structures

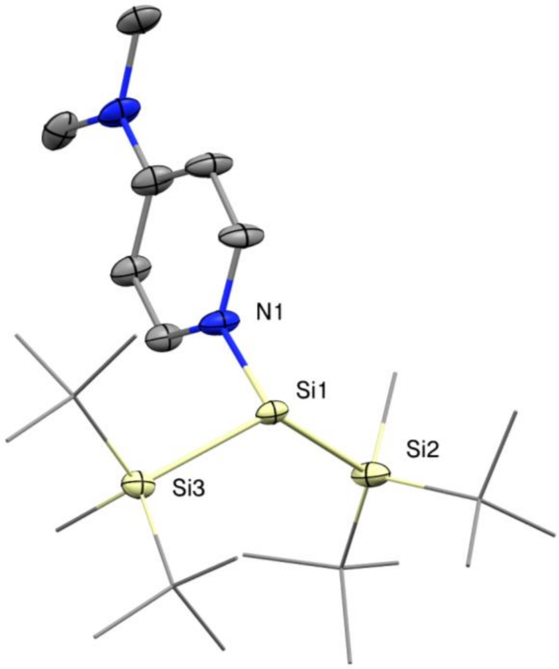

Fig. S20: SC-XRD structure of silylene **1b** with thermal ellipsoids drawn at the 30% probability level. Hydrogen atoms are omitted for clarity, ^{t}Bu- and Me-groups are simplified as wireframes. Selected bond lengths [Å] and angles [°]: Si1–N1 1.937(5), Si1–Si2 2.390(3), Si1–Si3 2.378(3), Si2–Si1–Si3 123.1(1), Si2–Si1–N1 96.2(2), Si3–Si1–N1 98.8(2).

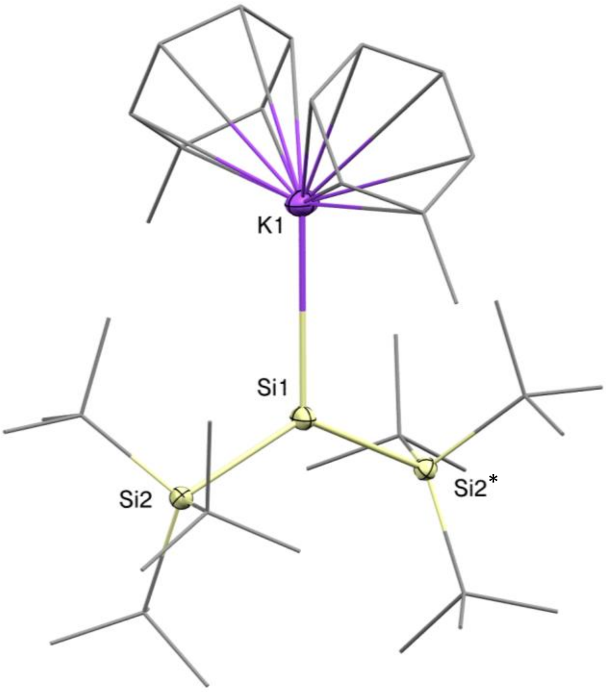

Fig. S21: SC-XRD structure of silyl radical **2** with thermal ellipsoids drawn at the 30% probability level. Hydrogen atoms are omitted for clarity, ^{t}Bu-groups and toluene molecules are simplified as wireframes. Selected bond lengths [Å] and angles [°]: Si1–Si2 2.3936(14), Si1–K1 3.315(2), K1–Si1–Si2 114.91(2), Si2–Si1–Si2* 130.19(3).

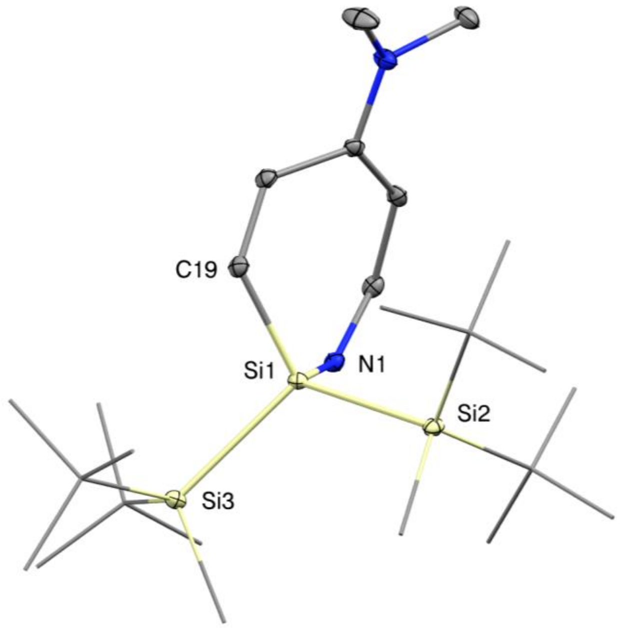

Fig. S22: SC-XRD structure of azasilepin **3** with thermal ellipsoids drawn at the 30% probability level. Hydrogen atoms are omitted for clarity, *ᵗ*Bu- and Me-groups are simplified as wireframes. Selected bond lengths [Å] and angles [°]: Si1–N1 1.750(1), Si1–C19 1.878(1), Si1–Si2 2.4144(6), Si2–Si1–Si3 113.74(2), Si2–Si1–N1 109.08(4), N1–Si1–C19 104.71(5).

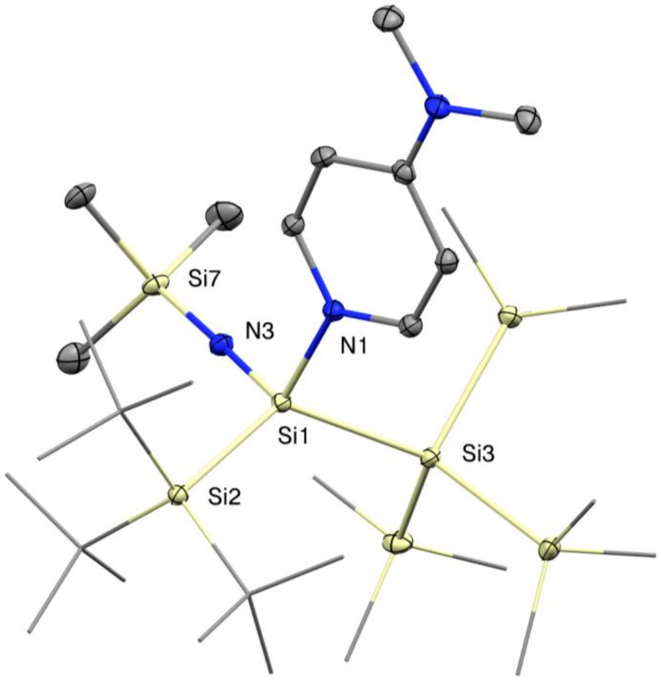

Fig. S23: SC-XRD structure of silaimine **7a** with thermal ellipsoids drawn at the 30% probability level. Hydrogen atoms are omitted for clarity, ^{t}Bu- and Me-groups are simplified as wireframes. Selected bond lengths [Å] and angles [°]: Si1–Si2 2.453(1), Si1–N1 1.928(2), Si1–N3 1.616(2), N3–Si7 1.660(2), Si2–Si1–Si3 125.08(3), N1–Si1–N3 106.08(8), Si1–N3–Si7 177.1(1).

2.3 Crystal data and structural refinement parameters

Table S1: Crystal data and structural refinement parameters for compounds **1**, **3**, **4** and **7a**.

Compound #	1b	2	3	7a
**CCDC #**	1967942	1967943	1967944	1967945
**Chemical formula**	$C_{25}H_{52}N_2Si_3$	$C_{38}H_{70}KSi_3$	$C_{25}H_{52}N_2Si_3$	$C_{31}H_{73}N_3Si_7$
**Formula weight**	464.96	650.31	464.96	684.55
**Temperature**	100(2) K	100(2) K	100(2) K	100(2) K
**Wavelength**	0.71073 Å	0.71073 Å	0.71073 Å	0.71073 Å
**Crystal size**	0.209 × 0.235 × 0.373 mm	0.176 × 0.299 × 0.302 mm	0.268 × 0.342 × 0.398 mm	0.059 × 0.153 × 0.213 mm
**Crystal habit**	clear dark red-brown fragment	clear yellow fragment	clear yellow fragment	clear yellow fragment
**Crystal system**	monoclinic	monoclinic	orthorhombic	triclinic
**Space group**	C 2/c	C 2/c	P b c a	P -1
**Unit cell dimensions**	a = 28.128(4) Å, α = 90°<br>b = 15.312(2) Å, β = 110.180(4)°<br>c = 14.6773(19) Å, γ = 90°	a = 20.678(7) Å, α = 90°<br>b = 12.877(5) Å, β = 120.067(10)°<br>c = 17.566(10) Å, γ = 90°	a = 11.9770(13) Å, α = 90°<br>b = 16.9418(17) Å, α = 90°<br>c = 28.631(3) Å, α = 90°	a = 11.515(9) Å, α = 97.728(17)°<br>b = 11.940(9) Å, β = 92.844(18)°<br>c = 16.100(7) Å, γ = 104.175(19)°
**Volume**	5933.4(14) Å^3	4048(3) Å^3	5809.6(11) Å^3	2119(2) Å^3
**Z**	8	4	8	2
**Density (calculated)**	1.041 g/cm^3	1.067 g/cm^3	1.063 g/cm^3	1.073 g/cm^3
**Absorption coefficient**	0.174 mm^{-1}	0.243 mm^{-1}	0.178 mm^{-1}	0.248 mm^{-1}
**F(000)**	2064	1436	2064	756
**Diffractometer**	Bruker D8 Venture Duo IMS	Bruker D8 Venture	Bruker D8 Kappa Apex II	Bruker D8 Venture Duo IMS
**Radiation source**	IMS microsource, Mo	TXS rotating anode, Mo	fine-focus sealed tube, Mo	IMS microsource, Mo
**Theta range for data collection**	1.94 to 25.35°	2.28 to 25.68°	2.20 to 26.37°	2.03 to 25.35°
**Index ranges**	-33<=h<=33, -18<=k<=18, -17<=l<=17	-25<=h<=25, -15<=k<=15, -21<=l<=20	-14<=h<=14, -21<=k<=21, -35<=l<=35	-13<=h<=13, -14<=k<=14, -18<=l<=19
**Reflections collected**	36869	68970	188411	89033
**Independent reflections**	5424 [R(int) = 0.0913]	3797 [R(int) = 0.0742]	5931 [R(int) = 0.0304]	7757 [R(int) = 0.0458]
**Coverage of independent reflections**	99.8%	98.6%	99.8%	99.9%
**Absorption correction**	Multi-Scan	Multi-Scan	Multi-Scan	Multi-Scan
**Refinement method**	Full-matrix least-squares on F^2	Full-matrix least-squares on F^2	Full-matrix least-squares on F^2	Full-matrix least-squares on F^2
**Refinement program**	SHELXL-2017/1 (Sheldrick, 2017)	SHELXL-2016/6 (Sheldrick, 2016)	SHELXL-2016/6 (Sheldrick, 2016)	SHELXL-2016/6 (Sheldrick, 2016)
**Function minimized**	$\Sigma w(F_o^2 - F_c^2)^2$	$\Sigma w(F_o^2 - F_c^2)^2$	$\Sigma w(F_o^2 - F_c^2)^2$	$\Sigma w(F_o^2 - F_c^2)^2$
**Data / restraints / parameters**	5424 / 6 / 328	3797 / 0 / 202	5931 / 0 / 287	7757 / 0 / 393
**Goodness-of-fit on F^2**	1.172	1.040	1.045	1.123
**Final R indices**	4238 data; I>2σ(I):<br>R1 = 0.1217, wR2 = 0.2651<br>all data: R1 = 0.1445, wR2 = 0.2780	3472 data; I>2σ(I):<br>R1 = 0.0305, wR2 = 0.0855<br>all data: R1 = 0.0341, wR2 = 0.0886	5356 data; I>2σ(I):<br>R1 = 0.0289, wR2 = 0.0771<br>all data: R1 = 0.0334, wR2 = 0.0802	7180 data; I>2σ(I):<br>R1 = 0.0402, wR2 = 0.0992<br>all data: R1 = 0.0439, wR2 = 0.1016
**Weighting scheme**	$w=1/[\sigma^2(F_o^2)+108.1245P]$<br>where $P=(F_o^2+2F_c^2)/3$	$w=1/[\sigma^2(F_o^2)+(0.0477P)^2+2.4242P]$<br>where $P=(F_o^2+2F_c^2)/3$	$w=1/[\sigma^2(F_o^2)+(0.0388P)^2+3.2781P]$<br>where $P=(F_o^2+2F_c^2)/3$	$w=1/[\sigma^2(F_o^2)+(0.0445P)^2+1.7142P]$<br>where $P=(F_o^2+2F_c^2)/3$
**Largest diff. peak and hole**	0.560 and -0.742 eÅ^{-3}	0.263 and -0.218 eÅ^{-3}	0.366 and -0.261 eÅ^{-3}	0.634 and -0.258 eÅ^{-3}
**R.M.S. deviation from mean**	0.104 eÅ^{-3}	0.040 eÅ^{-3}	0.042 eÅ^{-3}	0.058 eÅ^{-3}

3. References

[S1] G. R. Fulmer, A. J. M. Miller, N. H. Sherden, H. E. Gottlieb, A. Nudelman, B. M. Stoltz, J. E. Bercaw, K. I. Goldberg, *Organometallics* **2010**, *29*, 2176-2179.

[S2] T. Gross, H. Reinke, H. Oehme, *Can. J. Chem.* **2000**, *78*, 1399-1404.

[S3] A. Sekiguchi, T. Fukawa, M. Nakamoto, V. Y. Lee, M. Ichinohe, *J. Am. Chem. Soc.* **2002**, *124*, 9865-9869.

[S4] N. Wiberg, W. Niedermayer, H. Nöth, J. Knizek, W. Ponikwar, K. Polborn, D. Fenske, G. Baum, *Z. Anorg. Allg. Chem.* **2001**, *627*, 594-606.

[S5] D. Reiter, R. Holzner, A. Porzelt, P. J. Altmann, P. Frisch, S. Inoue, *J. Am. Chem. Soc.* **2019**, *141*, 13536-13546.

[S6] K. Fredenhagen, G. Cadenbach, *Z. Anorg. Allg. Chem.* **1926**, *158*, 249-263.

[S7] a) K. W. Klinkhammer, *Chem. Eur. J.* **1997**, *3*, 1418-1431. ; b) K. W. Klinkhammer, *Organosilicon Chemistry III - From Molecules to Materials* (Eds.: N. Auner, J. Weis), Wiley-VCH, **1997**.

[S8] *APEX suite of crystallographic software*, APEX 3 version 2015.5-2; Bruker AXS Inc.: Madison, Wisconsin, USA, 2015.

[S9] *SAINT*, Version 7.56a and SADABS Version 2008/1; Bruker AXS Inc.: Madison, Wisconsin, USA, 2008.

[S10] G. M. Sheldrick, SHELXL-2014, University of Göttingen, Göttingen, Germany, 2014.

[S11] C. B. Hübschle, G. M. Sheldrick, B. Dittrich, *J. Appl. Cryst.* **2011**, *44*, 1281-1284.

[S12] G. M. Sheldrick, SHELXL-97, University of Göttingen, Göttingen, Germany, 1998.

[S13] A. J. C. Wilson, *International Tables for Crystallography,* Volume C, Tables 6.1.1.4 (pp. 500-502), 4.2.6.8 (pp. 219-222), and 4.2.4.2 (pp. 193-199), Kluwer Academic Publishers: Dordrecht, The Netherlands, **1992**.

[S14] C. F. Macrae, I. J. Bruno, J. A. Chisholm, P. R. Edgington, P. McCabe, E. Pidcock, L. Rodriguez-Monge, R. Taylor, J. van de Streek, P. A. Wood, *J. Appl. Cryst.* **2008**, *41*, 466-470.
